# Impact of airborne particulate matter on skin: a systematic review from epidemiology to in vitro studies

**DOI:** 10.1186/s12989-020-00366-y

**Published:** 2020-07-25

**Authors:** Irini M. Dijkhoff, Barbara Drasler, Bedia Begum Karakocak, Alke Petri-Fink, Giuseppe Valacchi, Marc Eeman, Barbara Rothen-Rutishauser

**Affiliations:** 1 Adolphe Merkle Institute, University of Fribourg, Chemin des Verdiers 4, CH-1700 Fribourg, Switzerland; 2 Department of Biomedical and Specialist Surgical Sciences, University of Ferrara, Ferrara, Italy; 3 Department of Animal Sciences, PHHI NCRC, North Carolina State University, Kannapolis, NC USA; 4 Dow Silicones Belgium, Seneffe, Belgium

**Keywords:** Particulate matter, Air pollution, Urban pollution, Skin models, Skin, In vitro studies, In vivo studies, Oxidative stress, Inflammation, Barrier dysfunction

## Abstract

**Background:**

Air pollution is killing close to 5 million people a year, and harming billions more. Air pollution levels remain extremely high in many parts of the world, and air pollution-associated premature deaths have been reported for urbanized areas, particularly linked to the presence of airborne nano-sized and ultrafine particles.

**Main text:**

To date, most of the research studies did focus on the adverse effects of air pollution on the human cardiovascular and respiratory systems. Although the skin is in direct contact with air pollutants, their damaging effects on the skin are still under investigation. Epidemiological data suggested a correlation between exposure to air pollutants and aggravation of symptoms of chronic immunological skin diseases. In this study, a systematic literature review was conducted to understand the current knowledge on the effects of airborne particulate matter on human skin. It aims at providing a deeper understanding of the interactions between air pollutants and skin to further assess their potential risks for human health.

**Conclusion:**

Particulate matter was shown to induce a skin barrier dysfunction and provoke the formation of reactive oxygen species through direct and indirect mechanisms, leading to oxidative stress and induced activation of the inflammatory cascade in human skin. Moreover, a positive correlation was reported between extrinsic aging and atopic eczema relative risk with increasing particulate matter exposure.

## Introduction

Over 91% of the world’s population lives in areas of poor air quality with air pollutant concentrations exceeding the World Health Organization reference limits [1]. The State of Global Air 2019 report published by the Health Effects Institute (HEI) indicated that air pollution (Particulate Matter 2.5 (PM_2.5_), ozone, and household air pollution) is the fifth leading risk factor for mortality worldwide and in 2017, air pollution is estimated to have contributed to close to 5 million deaths globally [2].

Air pollutants can be grouped into gaseous pollutants (e.g., sulfur dioxide, nitrogen oxides, carbon monoxide, ozone, and volatile organic compounds), persistent organic pollutants (e.g., dioxins), heavy metals (e.g., cadmium, lead, mercury), and particulate matter (PM).

Among many sources, the primary source of PM is due to anthropogenic activities, arising from the combustion of fossil fuels used for transport and the generation of energy [3]. Traffic is one of the most significant contributors to urban PM [4], resulting in a ubiquitous air distribution. PM is a complex mixture, and the concentration, particle size, and chemical properties of PM vary widely in space and time. PM consists of primary particles, which are emitted directly to the atmosphere as a result of incomplete combustion processes or are produced by the abrasion of tires, of brakes, road surfaces, and the generation of fugitive dust. Additionally, PM contains secondary particles, which are chemically formed in the atmosphere from gaseous precursors [5]. Because of their fissured structure, these particles offer an ideal surface for the attachment of other (toxic) substances. According to the US Environmental and Protection Agency (EPA), PM can be classified according to particle size (diameter) as follows, PM_0.1_ (ultrafine particles, ≤ 0.1 μm), PM_2.5_ (fine particles, ≤ 2.5 μm), PM_10_ (coarse particles, ≤ 10 μm) [6].

The PM_10_ fraction consists primarily of crustal materials, sea salt, biological factors, including bacteria and fragments of pollen, and are generated by mechanical processes rather than combustion. On the other hand, PM_2.5_ and PM_0.1_ are predominantly produced by combustion processes and consist primarily of metals, hydrocarbons, and secondary particles formed by chemical reactions with gaseous compounds in the atmosphere [7, 8]. The majority of the particle mass is in a fraction of less than 2.5 μm, with the largest number of particles in the fraction in less than 0.1 μm. PM_0.1_ with the greater surface area can carry a large amount of adsorbed pollutants, oxygen gases, organic compounds, and transition metals [9]. An example of hydrocarbons that are frequently adsorbed on the PM’s surface is polycyclic aromatic hydrocarbons (PAHs) [10]. PAHs are considered to cause one of the most relevant health hazards due to their ability to induce the formation of reactive oxygen species (ROS) [11], as mediators of cardiovascular effects [12] and increases the risks of cancer in humans [13].

Long-term effects of air pollution on the pulmonary and cardiovascular system have been extensively studied, and include a strong correlation between different levels of air pollution and mortality, exacerbation of asthma, chronic bronchitis, respiratory tract infections, ischemic heart disease and stroke [14]. Although most of the attention of airborne PM has focused on the impact on human respiratory and cardiovascular systems [15, 16], many other primary and secondary organs (e.g., skin, gut, liver, kidney) are affected upon repetitive exposure to PM [17]. Indeed, because of its peculiar location, human skin acts as a biological shield against air pollutants, and prolonged and repetitive exposure to high levels of airborne PM has been shown to have profound adverse effects on cutaneous tissue [18–21].

This review highlights the current understanding of the impact of airborne PM on the skin and the underlying physiological mechanisms that are affected. Furthermore, it provides an in-depth overview of in vivo and in vitro studies of airborne PM exposure to skin and lists the biomarkers of interest and findings. The following search engines and databases, PubMed, ScienceDirect, Google Scholar, and Web of Science, were used to identify peer-reviewed research articles and reviews on the effects of airborne PM on skin. From among 540 articles identified, 75 articles were included for further consideration after manual screening of the articles. The articles that were excluded either did not contain data or were classified as editorials or conference abstracts. Finally, articles in languages other than English or German were also omitted.

## The structure and function of human skin

**Fig. 1:**
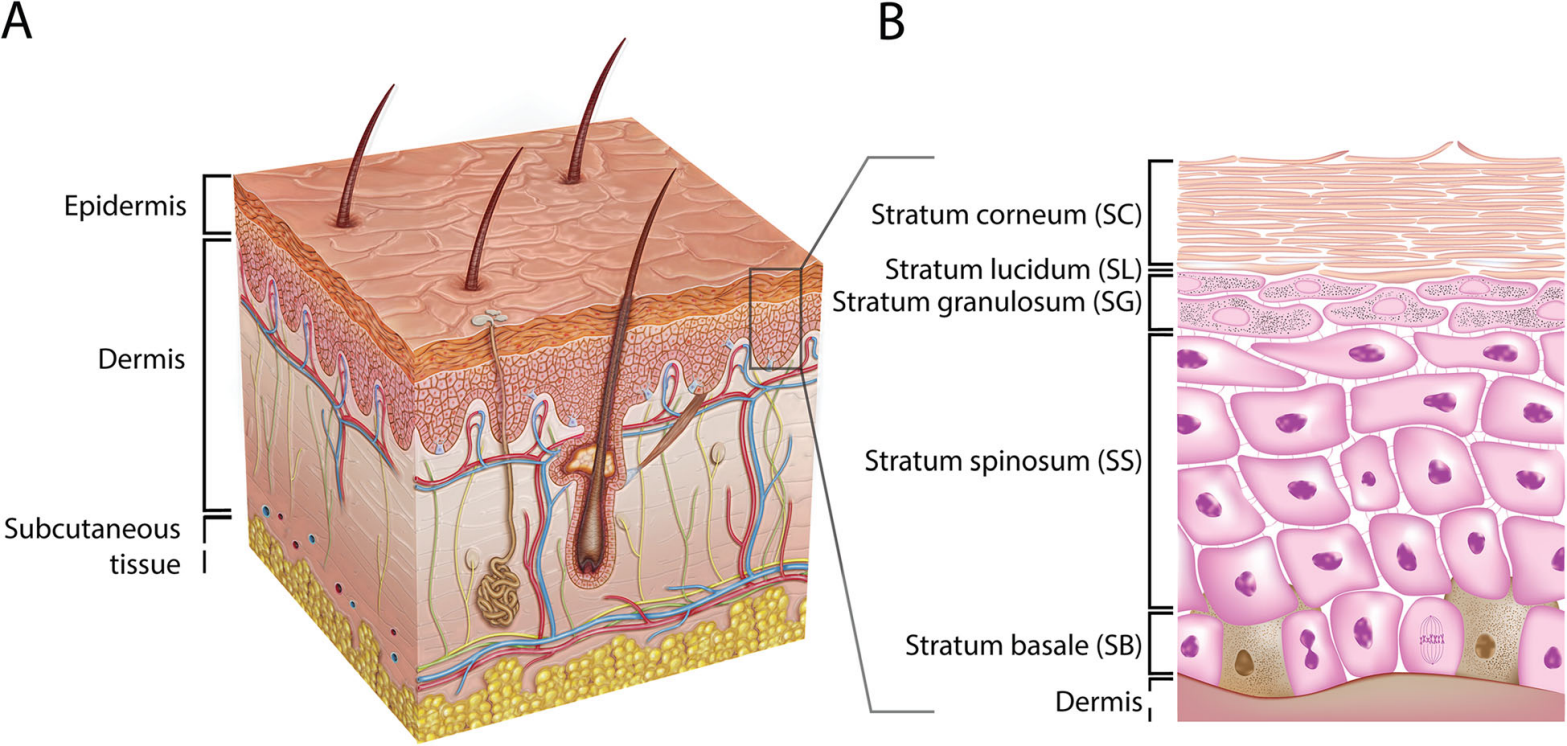
Schematic overview of the human skin. **(A)** The skin consists of the epidermis and the dermis. **(B)** The epidermal layer consists, from top to bottom, of the stratum corneum (SC), the stratum lucidum (SL), the stratum granulosum (SG), the stratum spinosum (SS), and the stratum basale (SB). Figure adopted with permission from van Smeden et al. [22]

Mammalian skin is the largest organ in the human body and can be divided into two major layers, the epidermis and the dermis (Fig. 1). The dermis, the lower layer of the skin, is made of connective tissue and has a thickness of up to 3 mm. Fibroblasts are the primary cell type in this layer [23, 24]. These cells secrete collagen and elastin, which contribute to the generation of the extracellular matrix, providing the dermis with strain resistance and elasticity, respectively [23, 25, 26]. Embedded within the dermis are structures such as nerve endings, sebaceous glands, hair follicles, and blood and lymphatic vessels [24]. The vascular network provides nutrients and oxygen to the upper skin layers and their surrounding tissues. Additionally, the vascular network takes part in detoxification, regulation of temperature, and wound repair. Besides fibroblasts, there are various other cell types found in the dermis: endothelial cells, mast cells, macrophages, dendritic cells called Langerhans cells, T cells, and neutrophils. Numerous dermal immune cells provide an effective defense mechanism against the invasion of pathogens and exogenous substances [27, 28].

The layer above the dermis, the epidermis, is divided into four to five layers and contains mainly keratinocytes [29]. The lower layer, the stratum basale (SB), is made of a single layer of undifferentiated keratinocytes. These cells are continually dividing and upon differentiation migrating up to the adjacent layer, the stratum spinosum (SS), where they start a maturation process, such as changing their shape from columnar to polygonal. The layer above the SS is the stratum granulosum (SG) that contains differentiated keratinocytes with a typical accumulation of cytoplasmic materials, e.g., keratohyalin granules. They also produce lamellar bodies (LBs) that secrete specific lipid compounds, which are essential for the formation of the uppermost layer of the skin, the stratum corneum (SC) [30, 31]. In thick skin, such as on the palms of the hands and soles of the feet, there is an additional thin layer named the stratum lucidum (SL), located between the SG and SC [32]. The SC is a multiple layered tissue (e.g., 15–20 layers), which generally has a thickness of 10–20 μm [33]. This layer consists of corneocytes that are terminally differentiated keratinocytes lacking both nucleus and cytoplasmic organelles [33–35], surrounded by a lipid matrix providing a permeability barrier. The described differentiation process is marked by the expression of early (e.g., cytokeratin 5 and 14) and late (e.g., cytokeratin 1 and 10) differentiation markers [36]. Other cells found in the epidermis are melanocytes, dendritic cells (i.e., Langerhans cells), T cells (e.g., CD8+ cells), and Merkel cells [29].

## Epidemiology

Exposure to airborne PM is associated with several skin diseases and disorders and has been extensively reviewed elsewhere [20, 21, 37–39].

In healthy skin, exposure to urban PM has been associated with accelerated extrinsic aging [40–42], its clinical symptoms being pigment spot formation, coarse wrinkle development, and elastosis [43]. Further evidence from epidemiological, clinical studies, and in vitro studies, and aging mechanisms have been reviewed [19, 44–46], all indicating the generation of free radicals, the activation of aryl hydrocarbon receptor signaling, the induction of inflammatory cascade, and finally the impairment of the skin barrier.

In Germany, a higher incidence of skin melanoma among women was reported for women living in rural areas compared to women living in non-rural areas [47]. In another semi-individual cohort study, an increase in PM_10_ resulted in a 52% increase in the relative risk of non-melanoma skin cancer [48]. The genotoxic potential of PM was further investigated in an in vitro study that showed (i) a positive dose-response relationship for PM-induced DNA reactivity, and (ii) the highest DNA reactivity for PM_2.5_ [7].

The chronic relapsing inflammatory skin disease, atopic eczema (AE), is characterized by skin barrier defects, intense pruritus, and immunological dysregulation [49–51]. This disease is not only caused by genetic predisposition (i.e., filaggrin mutation) and a dysregulated immune system but also triggered by several environmental factors, including air pollution, as suggested by recent epidemiological reports [20, 52]. A meta-analysis on skin diseases due to PM exposure included 13 studies and showed correlations between PM_10_ and PM_2.5_-exposure and several skin diseases [53]. It reported on a significant association between PM_2.5_-exposure of younger people (e.g., 2–30 years), which was not the case for PM_10_ exposure. However, with an odds ratio of 1.05 and the small quantity of observational, cohort studies, and individual studies, the statistical power of this meta-analysis is limited. Several new studies have been published that were not included in this meta-analysis [54–60]. More recently, Krämer and co-workers have reviewed this evidence by combining data from 57 environmental epidemiological studies in a systematic review [61]. No sufficient evidence for a higher AE incidence upon large-scale exposure assessments of air pollution (PM_10_ and sulfur dioxide (SO_2_)) was reported. Contrary to the small-scale exposure assessments (PM_2.5_), where in most of the studies (23 out of 30), a significant association between AE and traffic-related emissions was determined. Additionally, in all of the panel studies, there was a strong correlation between the AE symptom severity and outdoor pollution. Interestingly, an association between maternal exposure to traffic-related pollution and AE incidence in the offspring was found. From the prospective birth cohort study, Cohort for Childhood Origin of Asthma and Allergic Diseases (COCOA) [62], 468 one-year-olds were included with measurements of outdoor PM_10_ and PM_2.5_ and transepidermal water loss (TEWL), which is a measure for skin barrier dysfunction [63]. The effects of prenatal PM exposures were studied during three trimesters on skin barrier dysfunction and AE [64]. A positive correlation was found between prenatal PM exposure during the first trimester and the skin barrier dysfunction and AE in the offspring: early-onset AE and higher AE severity at age three. It is suggested that PM-induced oxidative stress could induce epigenetic changes in fundamental DNA repair genes in the placenta, which could affect fetal immune development. Pulmonary and intranasal exposure to PM_10_, PM_2.5_, and PM_0.1_ have been extensively studied for their ability to enhance Th2-related immune responses, their role as an immune adjuvant, and their effects on the development of atopic disease [65–67].

Dong and co-workers reviewed reports on PM exposure and skin inflammation in China and concluded that, indeed, PM emissions aggravate the symptoms of inflammatory skin diseases like AE [68]. The aforementioned results have been supported by a recent review on the impact of different air pollutants on AE, which included a total of 21 in vitro studies, animal studies, clinical trials, and case studies [69].

The exact mechanisms through which PM can aggravate AE symptoms are yet to be further investigated. Additionally, determining the synergistic effects of different air pollutants while taking solar radiation into account can be challenging and is often not addressed when studying the effect of a single pollutant on the skin [70]. In a study from Japan, biopsies from 75 AE patients were investigated and a correlation was found between several oxidative stress markers in the SC and the severity of the disease [71]. It is hypothesized that environmentally generated reactive oxygen species can induce oxidative damage to proteins in the SC. Eventually, this may lead to the disruption of the skin barrier and further exacerbation of AE. Additionally, the aryl hydrocarbon receptor may play a key role in PM-induced AE aggravation. It is suggested that PM and AE are linked by the activation of the aryl hydrocarbon receptor signaling pathway and transactivation of neurotrophic factor artemin, by ligand species that are adsorbed onto airborne PM [72, 73].

Thus far, the studies mostly investigated the link between AE and PM. However, it is hypothesized that patients with other inflammatory skin diseases with an impaired barrier function, such as the rare genetic skin disease Netherton syndrome [74] and psoriasis [75], are more prone to the effects of PM.

## Simulating biological responses of the skin to airborne PM in vivo and in vitro

The effects of airborne PM on the skin and underlying mechanisms will be critically evaluated, i.e., by reviewing studies on exposure of in vitro human two-dimensional (2D) and three-dimensional (3D) models, and finally in vivo animal models. The studies used either standard reference materials or in-house diesel emission and urban particulate matter extracts as exposure constituents.

### Airborne PM for experimental studies

As aforementioned, the composition of PM is complex and includes metals, organic compounds (e.g., organic carbon and materials of biological origin), inorganic carbonaceous material, sulfate, and nitrate, among others. To assess their health effects, different approaches have been applied, such as epidemiological studies, controlled exposure studies to a defined source, or collection of particles on filters which can then be used for further experiments. It is important to add that the thorough characterization of the particles, including particle size and chemical analysis of the adsorbed chemicals onto the particles to correlate observed effects, is challenging but highly relevant [5].

The most common particles used in in vitro and in vivo studies to simulate the effects of airborne PM on the skin are Standard Reference Materials® available from the National Institute of Standards and Technology (NIST) [76]. NIST offers three different diesel PM extracts: SRM® 1650b, SRM® 1975, and SRM® 2975. Both SRM® 1975 and SRM® 2975 contain diesel PM collected from a filtering system of a diesel-powered forklift engine. SRM® 1650b contains material that is obtained from the heat exchangers of a dilution tube connected to diesel engines. This standard reference material is considered representative of particulate emissions of heavy-duty diesel engines and can be categorized as PM_2.5_. Besides using certified standards, diesel particulate extracts from several different diesel engine emissions collected on filters have been extracted and tested on skin models. Additionally, NIST offers two airborne PM standards named SRM® 1649b and SRM® 1648a that have been collected in the Washington, DC area and the St. Louis, MO area, respectively, between 1976 and 1977. Other standards for fine atmospheric PM are SRM® 2787 (i.e., PM_10_) and SRM® 2786 (i.e., PM ≤ 4 μm) collected in Prague, the Czech Republic in 2005.

The Joint Research Centre (JRC) of the European Commission produces European Reference Materials (ERM) that are used to simulate airborne PM [77]. JRC is accredited to ISO 17034, ensuring the appropriate quality of ERMs. They offer ERM-CZ100 and ERM-CZ120 fine urban dust particles that are processed in a way that resembles PM_10_-like urban dust without (e.g., ERM-CZ120) or with PAHs (ERM-CZ100). The National Institute for Environmental Studies (NIES) in Japan offers PM_10_-like urban dust certified reference material (CRM) no. 28 that have been collected from a central ventilating system in a building in Beijing, China [78].

Several studies reported self-collection of airborne PM or PM from seasonal dust storms in Asia and West Africa, e.g., on building rooftops in Asia, using an in-house filtering system, yielding PM_2.5_. Others reported usage of concentrated ambient air particles (CAPs), which are airborne particles that are concentrated to PM_2.5_ for skin model exposure.

### Skin models used to study airborne PM effects

#### Animal models

The main in vivo approaches to study skin responses to either drug permeation or toxicological outcome are either rodents (i.e., rats or mice) or pigs [79]. Rodent skin differs significantly from human skin, due to considerably thinner skin layers and a higher hair follicle density, leading to higher substance permeation [80]. On the contrary, porcine skin more closely resembles the structural properties of the human skin [81–84]; however the animals are more challenging to handle and have higher fat storage [85].

Animal models are employed to compare PM effects on skin with a healthy and compromised barrier function. A disrupted barrier can be created by the conventional tape stripping method, which consists essentially of removing the SC of the animal skin model layer by layer [86–92]. Another approach is to use the NC/Nga mouse model that develops skin lesions that are comparable to human AE lesions [93–96].

Animal models have various disadvantages, besides their different physiological structures. Animal studies are highly time and cost-ineffective; they are restricted to many regulations and countless ethical concerns [97]. To effectively comply with and implement the three R’s (i.e., reduction, refinement, and replacement) [98] for the laboratory use of animals, alternatives to animal testing like 2D and 3D in vitro human skin models should be considered to comply with the reduction and replacement concept [99].

#### In vitro human 2D models

Human adult low calcium high temperature (HaCaT) cells are an immortalized keratinocyte cell line that is frequently used for 2D in vitro studies [100]. However, a significant limitation of this cell line is their limited response to major T helper cell cytokines [101]. It was shown that HaCaT cells respond differently to these pro-inflammatory cytokines compared to primary cells, resulting in different expression profiles of genes related to the development of the skin barrier [102]. Therefore, a model using HaCaT cells that focuses on the pathophysiological response towards pro-inflammatory cytokines should necessitate careful interpretation. The cells that are considered more biologically relevant are primary cells, which are explanted directly from healthy donors. The primary skin cells that are most often studied concerning the effects of PM are normal human epidermal keratinocytes (NHEK) or normal human dermal fibroblasts (NHDF) monocultures. Human primary cells are either isolated from the neonatal foreskin or adult skin. In a study that investigated the proteome in primary keratinocytes with respect to gender and age, it was determined that there were only minor differences [103]. Primary skin cells are cultured on specific substrates in an optimized cell culture medium and receive PM treatment directly in their culture medium, referred to as systemic treatment. Although the use of 2D models allows for easy, fast, and low-cost experiments, they lack a physiological skin barrier with a competent SC layer. Without this protective layer, the viable cells are in immediate contact with the tested substance. Therefore, systemic treatment of a 2D model consisting of living cells could result in an overestimation of the impact of the testing material and not resemble the real-life exposure situation. It was found that the keratinocytes cultured in 3D, even upon a systemic treatment, are more resistant to cytotoxic agents such as hydrogen peroxide compared to keratinocytes cultured in 2D [104]. A recent study compared the effect of silver nanoparticles on oxidative stress and inflammation in 2D and 3D skin models [105]. Cell viability was reduced, and the silver content was approximately 9-fold higher in 2D keratinocytes compared to a 3D model. Additionally, the oxidative stress and pro-inflammatory response were substantially stronger in a 2D model compared to a 3D model. For this reason, the use of a 3D skin model to better simulate the real-life topical exposure condition and assessment of the skin’s barrier function upon the treatment is recommended.

#### In vitro human 3D models

The two main types of 3D models used to mimic human skin are human epidermal equivalents (HEE) and human skin equivalents (HSE) [106, 107].

Cultivation of HEE models starts with one cell type: either primary keratinocytes isolated from the human epidermis, or immortalized cell lines (e.g., N/TERT). These keratinocytes are grown in typical culture inserts on a supporting substrate, such as an inert membrane with a defined pore size or collagen matrix, using the air-liquid culture technique. This technique includes removing the cell culture medium from the upper side to expose the cells to air on one side, allowing the cells to ‘feed’ from the medium in the chamber underneath [108–110]. Upon stimulation with growth factors, insulin, ascorbic acid, and calcium, these keratinocytes can terminally differentiate into corneocytes, forming the upper layers of the epidermis over a time span of one to two weeks.

HSE models are comprised of both a dermal and an epidermal compartment consisting of fibroblasts embedded in a collagen matrix and keratinocytes, respectively. This two-compartmental model creates an interplay between the two skin layers, resulting in a better resemblance to human skin.

Although 3D skin models like the HSE and HEE are practical models that well-resemble the human skin, their barrier properties are less defined, due to a different SC lipid composition and a higher barrier permeability compared to that of native human skin [80, 111–115]. In 3D models exposed to the air-liquid interface, PM can be applied either directly in the culture medium (systemic treatment) or on the topical side of the skin model either as aerosol or suspension (topical treatment). Topical treatment is considered more physiologically relevant compared to real-life exposures as opposed to systemic treatment.

## Effects of PM in vivo and in vitro

### PM-induced skin barrier dysfunction and particle penetration

There are four different barrier compartments in the epidermis: physical, microbial, chemical, and immunological compartments [106]. In this section, the effects of PM on the physical, microbial, and chemical barrier compartments will be reviewed.

#### Microbial barrier

The microbial barrier compartment consists of a diverse range of microorganisms, called the commensal skin microbiome that colonizes the skin. This includes colonization by millions of bacteria, fungi, and viruses, and the composition varies depending on the location of the skin [116]. These commensals protect the skin by preventing infection by pathogenic microbes. Although the research field of the skin’s microbiome has been given rise to many investigations over the last years [116, 117], studies on PM and the skin’s microbiome are scarce. It was shown that the composition of children’s skin microbiome was dependent on their age and living environment (e.g., rural versus urban), indicating a possible link between PM exposure and the skin microbiome composition [118]. Nevertheless, the role of the microbial barrier in PM-induced damages remains yet to be unraveled.

#### Physical barrier

Reported findings on the effects of PM on the skin’s barrier function in vivo and in vitro are summarized (Table 1).

**Table 1:** Review of reported effects on the barrier function, such as proliferation, differentiation, and PM penetration in varying skin models upon exposure to PM

Model	PM type	Dose and application	Exposure time	Main findings	Ref.
Ex vivo human skin	SRM® 1649b	2 mg/cm^2^, topical	24 h	- Pyknotic nuclei. - Decreased collagen-1 protein expression.	[119]
Pig	SRM® 1648a and 1649b	100 μg/m^2^, topical	5 d	- Decreased E-cadherin, cytokeratin, and filaggrin protein expression.	[120]
Mice, BALB/c with disrupted barrier	PM ≤1 μm from Seoul, Korea	8 μg/cm^2^, topical	6 h and 2 w repetitive exposures, 10 times total	- Healthy barrier: PM remained in follicles. - Disrupted barrier: penetration of PM in SS. - Repeated exposure: dermal penetration of PM.	[121]
Mice, BALB/c	SRM® 1649b	100 μg/m^2^, topical	24 h repetitive exposures, 5 d total	- Decreased filaggrin protein expression.	[122]
Mice, HR-1	SRM® 1650b	100 μg/mL, topical	7 d	- PM internalization.	[123]
	SRM® 1650b	100 μg/mL, topical	7 d	- Increased keratin 10 and PCNA protein expression.	[124]
HSE	SRM® 2975	200 μg/mL, systemic	2 d repetitive exposures, 6 d total	- Decreased keratin 16, keratin 17, and Ki67 protein expression.	[125]
	ERM-CZ120	200 μg/mL, systemic	48 h	- Altered morphology of epidermis.	[126]
HEE	Diesel PM or vapor	0.05% (v/v), systemic	20 d	- Decreased protein expression of cornifin A, suprabasin, and antileukoproteinase.	[127]
	CAPs, PM_2.5_	0.5 and 2.0 μg/cm^2^, topical	24 and 48 h	- PM penetration in SC after 24 h. - PM penetration in deeper layers after 48 h. - No alterations in morphology.	[128]
	CRM no. 28	25 mg, topical	6 h	- *Claudin 4* gene expression is upregulated. - No changes in mRNA expression of *filaggrin*, *loricrin*, *involucrin*, *transglutaminase 1*, *keratin 1*, *keratin 10*, *keratin 5*, and *keratin 14*.	[129]
	SRM® 1648a	200 ppm, systemic	96 h	- Altered morphology. - Decreased PCNA and filaggrin protein expression.	[130]
	SRM® 1648a	2.2, 8.9, and 17.9 μg/cm^2^, topical	24 and 48 h	- Decreased cytokeratin 10, involucrin, and loricrin protein expression. - Increased barrier permeability for 17.9 μg/cm^2^. - Increased protein expression of aquaporin 3.	[131]
	PM_2.5_ from Seoul, Korea	50 μg/mL, topical	24 h	- Decreased keratin-10, claudin-1, desmocollin-1, and IFN-γ protein expression. - Increased S100A7 and S100A8 protein expression.	[132]
	PM_0.3–2.5_ from Benin, West-Africa	30 μg/cm^2^, topical	24 h	- Decreased loricrin protein expression.	[133]
	PM_2.5_ from Xi’an, China	50 μg/mL, systemic	24 h repetitive exposures, 2, 4, or 6 d total	- Increased cholesterol levels. - Reduced squalene levels.	[134]
NHDF	SRM®2787	30 μg/cm^2^, systemic	24 h	- PM internalization into autolysosomes.	[135]
	ERM-CZ100	200 μg/mL, systemic	24 h	- Increased elastase and collagenase activity. - Increased procollagen protein expression.	[136]
	The pre-conditioned medium of HaCaT treated with CRM no. 28	125 μg/mL, systemic	30 m, 48 h post-incubation	- Increased elastase and collagenase activity.	[137]
NHEK	PM_2.5_ from Xi’an, China	50 μg/mL, systemic	24 h	- Top upregulated genes of transcriptomics analysis are *S100A8*, *S100A9*, *keratin 6B*, *keratin 19*, *serpin B3*, and mostly the genes are involved in cholesterol metabolism.	[134]
	Diesel PM or vapor	0.05% (v/v), systemic	20 d	- Decreased envoplakin, suprabasin, filaggrin, involucrin, sciellin, caspase-14, cornifin-A protein, keratin-1, and keratin-10 protein expression.	[127]
	Diesel PM	30 and 60 μg/mL, systemic	24 h	- PM internalization.	[138]
	Asian dust storm particles from Seoul, Korea	25 μg/mL, systemic	24 h	- Increased *caspase 14* and *ITGA6* mRNA expression.	[139]
	PM ≤1 μm from Seoul, Korea	40 μg/cm^2^, systemic	24 h	- PM internalization.	[121]
	PM_2.5_ from Seoul, Korea	50 μg/mL, systemic	2 or 4 d	- Decreased number and length of cilia. - Increased SPRR3 protein expression. - Decreased *keratin 1*, *keratin 10*, and *IFT88* mRNA expression.	[140]
	SRM®2786	1 mg/mL, systemic	6 h	- RNA-Seq analysis: Upregulation of *keratin-associated protein 2–3*, *keratin 13*, *keratin 34*, and *claudin 4*. Downregulation of *growth differentiation factor 15*.	[141]
	PM_2.5_ from Seoul, Korea	25 μg/mL, systemic	24 h	- Top upregulated genes from transcriptomics analysis are *SPRR* family members and other cornified envelope-related genes, *transglutaminase 3*, *involucrin*, *filaggrin*, *keratin 10*, *keratin 15*, *S100A7*, *S100A8*, *S100A9*, and *S10012*.	[132]
HaCaT	CRM no. 28	125 μg/mL, systemic	30 m, 24 h post-incubation	- Increased elastase and collagenase activity.	[137]
	SRM® 1649b	50 μg/cm^2^, systemic	24 h	- Decreased aquaporin-3 protein expression.	[142]
	SRM® 1649b	50 μg/cm^2^, systemic	24 h	- Decreased filaggrin, repetin, involucrin, and loricrin protein expression after 24 h.	[143]
	PM_2.5_ from Shanghai, China	10–100 μg/mL, systemic	24 h	- Increased involucrin, repetin, and filaggrin (highest dose) protein expression. - No changes in loricrin protein expression.	[144]
	SRM® 1649b	25 and 50 μg/cm^2^, systemic	4 and 24 h	- Decreased filaggrin protein expression.	[122]
	SRM® 1650b	50 μg/mL, systemic	1, 4, 8, 12, and 24 h	- PM internalization.	[123]
	SRM® 2975	100 and 200 μg/mL, systemic	24 h	- No changes in loricrin protein expression. - Increased involucrin protein expression. - Decreased keratin 16, keratin 17, and Ki67 protein expression. - Decreased BrdU incorporation.	[125]
	CAPs, PM_2.5_	5–25 μg/mL, systemic	1, 3, 6, and 24 h	- PM internalization.	[145]

Abbreviations: w, week; d, day; h, hour; m, minute; HSE, human skin equivalent; HEE, human epidermal equivalent; NHEK, normal human epidermal keratinocyte; SRM, standard reference material; PM, particulate matter; CAP, concentrated ambient particles; SS, stratum spinosum; ppm, parts per million; CRM, certified reference material; BrdU, 5-bromo-2′-deoxyuridine

The physical barrier compartment of the skin can be seen as a so-called “outside-in” and “inside-out” barrier. The “outside-in” barrier consists of the SC, which protects against the penetration of pathogens, allergens, and other exogenous substances such as PM [146, 147]. Several distinct proteins in the epidermis are crucial to the terminal differentiation program and the barrier function of the skin, such as involucrin, loricrin, and profilaggrin [148, 149]. Keratin filaments in the SG keratinocytes interact with profilaggrin, leading to aggregation of these filaments. Additionally, the intracellular components are degraded, and the desmosomes are transformed into corneodesmosomes, linking the corneocytes together that results in a densely packed layer [150]. The cornified envelope is formed around the plasma membrane and composed of structural proteins like involucrin and loricrin that are cross-linked by calcium-dependent transglutaminases [147]. Another protein that is crucial to the cornified envelope structure is the members of the small proline-rich (SPRR) family. SPRR3 was shown to be upregulated in keratinocytes treated with PM_2.5_ [132]. SPRR3 upregulation has been shown to decrease ciliogenesis in vitro [140], which is responsible for suppressing skin pigmentation in melanocytes [151].

The “inside-out” barrier, mainly provided by tight junctions, prevents excessive TEWL and, therefore, protects the skin against dehydration [146, 147, 152]. It is likely that PM disrupts the barrier function of the skin, by modulating or even degrading the tight junctions. In other epithelial cells like lung cells or nasal cells, PM is shown to disrupt the barrier by modulating and even degrading tight junction proteins such as occludin, zona occludens-1 (ZO-1), and claudin-1 [153, 154]. Also, increased endothelial barrier permeability was shown to be PM-induced, due to ZO-1 degradation [155].

In animal models, PM treatment mainly involved repetitive topical treatment for a longer time period (up to 2 weeks) of a relatively high dose (0.1–8 mg/m^2^), resulting in decreased expression of structural barrier protein like keratins and filaggrin [120–122]. In porcine skin, deterioration of the skin barrier by PM due to disrupted tight junctions led to increased skin permeability [120]. In mice, penetration of PM was observed in both healthy skin and to a greater extent in the skin with a decreased barrier function (e.g., tape-stripped skin or skin of an AE mice model) [121]. In 2D cultures, e.g., in keratinocytes and fibroblasts, cellular internalization of PM was observed after short-term exposures (i.e., 24 h) [121, 123, 135, 138, 145]. It is important to note that engineered nanoparticles (e.g., single particles with a diameter less than 100 nm [156]) have been reported to penetrate the first layers of the epidermis (i.e., SC and SG) and few reports on penetration into deeper layers of the viable epidermis or even dermal layers [157, 158]. However, the penetration of PM depending on size and agglomeration into the epidermis in healthy and diseased skin must be evaluated in future studies.

In 3D HSE models, a systemic treatment with PM (200 μg/mL) induced alterations in skin morphology and expression levels of structural proteins after a long time exposure (i.e., 48 h up to 6 days) [125, 126]. In 3D HEE models, PM usually in concentrations of up to 50 μg/mL was mainly applied topically and short-term exposures (i.e., 24 to 48 h) were performed [128, 130, 132, 133]. PM was shown to affect the physical epidermal barrier in 3D models, as indicated by altered expression of proteins important for epidermal differentiation, such as filaggrin, involucrin, loricrin, and keratins [125, 127, 129, 130, 132, 133]. Additionally, markers for proliferation, such as Ki67, 5-bromo-2′-deoxyuridine (BrdU) incorporation, and proliferating cell nuclear antigen (PCNA), were shown to be decreased upon PM exposure [125, 130]. Reduced expression of differentiation and proliferation markers was also shown in 2D models, i.e., HaCaT and NHEK cells [122, 125, 127, 140, 141, 143, 144].

PM was also reported to affect the skin’s cholesterol metabolism and increased cholesterol levels in 2D and 3D skin models [134]. Cholesterol is one of the main lipid components of the matrix surrounding the corneocytes in the SC, thereby actively contributing to the permeability barrier on the epidermis. Altered cholesterol metabolism in the skin due to PM exposure could indicate an altered barrier function of the skin.

#### Chemical barrier

Keratinocytes are crucial to the production of antimicrobial peptides (AMPs), serving as the chemical barrier compartment, preventing microbial invasion at sites of damaged epithelium by directly terminating the pathogen. These molecules can destroy bacteria either by creating holes in their cellular walls or by sequestering iron, a crucial nutrient for bacterial growth [159]. Examples of AMPs are cathelicidin (i.e., LL-37) [160], defensins, and the S100 protein family [161]. The expression of numerous genes that are encoding AMPs is differentially regulated by several pro-inflammatory cytokines [162]. Increased AMP expression was observed in a 3D HEE and primary keratinocytes treated with PM [132, 134], indicating an alteration in the chemical epidermal barrier.

### PM-induced oxidative stress

One of the primary mechanisms of the adverse effects of PM is through the generation of ROS. ROS are a group of highly reactive chemical species that are derived from the oxygen metabolism characterized by an unpaired electron (e.g., hydroxyl radical and superoxide anion) [163]. When the production of ROS overwhelms the skin’s antioxidant defense, tissues incur an oxidative stress condition [164]. ROS can damage lipids, proteins, and DNA, leading to oxidative injury via stress on several cellular organelles, resulting in tissue damage [165]. Reports on PM-induced oxidative stress in the skin have been reviewed and summarized (Table 2). In brief, PM can induce ROS generation via direct and indirect mechanisms, as further discussed in this section.

**Table 2:** Review of reported effects on oxidative stress in varying skin models upon exposure to PM

Model	PM type	Dose and application	Exposure time	Main findings	Ref.
Ex vivo human skin	SRM® 1649b	2 mg/cm^2^, topical	24 h	- Lipid peroxidation.	[119]
Mice, C-57	Diesel PM	4.5, 11.1 and 26.7 mg/cm^2^, topical	80 h	- Increased DNA adduct formation.	[166]
Mice, FVB/N	SRM® 1650b	1 mg/time (PAH extracted), topical	12 h	- Increased *CYP1B1* mRNA expression.	[167]
Mice, HR-1	SRM® 1650b	100 μg/mL, topical	7 d	- Increased protein carbonylation and lipid peroxidation.	[168]
	SRM® 1650b	100 μg/mL, topical	7 d	- ER stress: upregulation protein expression of CHOP and GRP78. - Lipid peroxidation: increased expression of HNE protein. - Mitochondrial and ER swelling. - Increased protein carbonylation. - Apoptosis: increased protein expression of BAX, active caspase-3, and caspase-9, and DNA breakage. - Autophagy: increased protein expression of LC3B-II.	[123]
	SRM® 1650b	100 μg/mL, topical	7 d	- Increased protein carbonylation. - Increased NOX4 protein expression.	[169]
HSE	SRM® 2975	200 μg/mL, systemic	2 d repetitive exposure, 6 d total	- Increased protein expression of cleaved caspase-3.	[125]
HEE	CAPs, PM_2.5_	0.5 and 2.0 μg/cm^2^, topical	24 and 48 h	- Increased isoprostanes protein level. - Increased HNE protein expression. - Increased CYP1A1 protein expression. - Increased DNA fragmentation.	[128]
	CAPs, PM_2.5_	25 μg/mL, topical	24 and 48 h	- Increased protein carbonylation.	[170]
	PM_0.3–2.5_ from Benin, West-Africa	30 μg/cm^2^, topical	24 h	- Increased HNE protein expression. - Increased *HMOX1*, *metallothionein 1G* and *1E, cyclin dependent kinase inhibitor 2A,* and *caspase 3* mRNA expression. - Decreased *BIRC5* mRNA expression.	[133]
	CRM no. 28	25 mg, topical	6 h	- Upregulated mRNA expression of *CYP1A1*, *CYP1B1*, and *SOD2*.	[129]
	SRM® 1975	5 mg/mL, topical	48 h	- Increased level of carbonylated proteins	[171]
	SRM® 1648a	2.2, 8.9, and 17.9 μg/cm^2^, topical	24 and 48 h	- Decreased AhR and increased NOTCH1 protein expression.	[131]
NHDF	SRM® 2787	30 μg/cm^2^, systemic	24 h	- Autophagy: accumulation of LC3-II. - Mitochondrial stress: deformed mitochondria. - Increased *CYP1A1* and *CYP1B1* mRNA expression.	[135]
	ERM-CZ100	50–400 μg/mL, systemic	3.3 h	- Increased levels of intracellular ROS.	[136]
	The pre-conditioned medium of HaCaT treated with CRM no. 28	125 μg/mL, systemic	30 m, 48 h post-incubation	- Increased levels of intracellular ROS. - Increased number of apoptotic bodies.	[137]
	SRM® 1649b	50 μg/mL, systemic	24 h	- Increased intracellular ROS levels. - Activation of AhR (XRE activity). - Upregulation of *CYP1A1* mRNA expression.	[119]
	SRM® 1649b	100–400 μg/mL, systemic	24 h	- Nuclear translocation of AhR. - Increased mRNA expression of *CYP1A1*. - Induced apoptosis.	[172]
NHEK	Diesel PM or vapour	0.05% (v/v), systemic	20 d	- Increased Nrf2 protein expression. - Mitochondrial dysfunction: overexpression of proteins from mitochondrial complex I and IV.	[127]
	SRM® 1975	5 mg/mL, systemic	1 and 24 h	- Increased intracellular ROS levels. - Increased of *CYP1A1* mRNA expression. - Nuclear translocation of AhR.	[171]
	ERM-CZ120	3, 10, 30 and 100 μg/mL, systemic	24 and 48 h	- Increased ROS levels after 24 h. - Increased *NOX1* and *NOX2* mRNA expression after 24 h.	[173]
	PM ≤1 μm from Seoul, Korea	40 μg/cm^2^, systemic	24 h	- Increased ROS production. - Inhibition of ROS inhibited cytokine secretion.	[121]
	PM_2.5_ from Seoul, Korea	25 μg/mL, systemic	24 h	- Top upregulated genes from transcriptomics analysis are *CYP1A1* and *CYP1B1.*	[132]
	PM_2.5_ from Xi’an, China	50 μg/mL, systemic	24 h	- Top upregulated genes from transcriptomics analysis are *CYP1A1* and *SOD2*.	[134]
	Asian dust storm particles from Seoul, Korea	25 μg/mL, systemic	24 h	- Increased *CYP1A1*, *CYP1A2*, and *CYP1B1* mRNA expression.	[139]
	Diesel PM	30 and 60 μg/mL, systemic	24 h	- Increased ROS production. - Increased *HMOX1* mRNA and protein expression. - Increased *Nrf2* mRNA expression.	[138]
	SRM® 2786	1 mg/mL, systemic	6 h	- RNA-Seq analysis: Downregulation of ER stress apoptosis-related genes such as *ATF4* and *CHOP*. No activation of *BCL2*, *BAX*, *caspase 3*, and *caspase 8*. - Upregulation of *HMOX1*, *CYP1A1*, *CYP1B1*, and *NQO1*.	[141]
	SRM® 1650b and 2975	10 and 100 μg/mL, systemic	1 and 24 h	- Increased radical production.	[174]
NHEK, HaCaT, and HEK001	SRM® 1650b	50 μg/mL, systemic	72 h	- Induced senescence: increased β-galactosidase activity.	[124]
NHEK and HaCaT	SRM® 1650b	50 μg/mL, systemic	0.5–48 h	- Increased intracellular ROS levels. - Nuclear translocation of AhR (0.5 h). - Induced senescence: upregulation of P16^INK4A^ and increased number of SAHF/nuclei. Decreased colony-forming ability. - Senescence is AhR dependent. - Transcriptional regulation of P16^INK4A^ correlates with DNA demethylation: lower methylation of the P16^INK4A^ promoter region.	[124]
NHDF and HaCaT	SRM® 1650b	50 μg/mL, systemic	30 m and 24 h	- Increased levels of lipid peroxidation and protein carbonylation after 24 h. - Increased levels of superoxide anion, hydroxyl radicals, and intracellular ROS (30 m). - Increased intracellular and mitochondrial calcium levels after 24 h. - Increased protein expression of CHOP, GRP78, active caspase-3, caspase-9, PARP, and BAX after 24 h. - Downregulated protein expression of Bcl-1 and Mcl-1 after 24 h. - Increased mitochondrial permeability after 24 h. - Reduced ATP levels after 24 h. - Increased DNA degradation and the number of apoptotic bodies after 24 h.	[175]
HaCaT	PM_2.5_ from Bangkok, Thailand	100 μg/mL, systemic	30 m	- Increased intracellular ROS levels.	[176]
	PM_2.5_ from Taoyuan, China	25, 50, 100 and 200 μg/mL, systemic	24 h	- Increased intracellular ROS levels. - Decreased SOD activity. - Increased lipid peroxidation: accumulation of MDA protein. - Induced formation of apoptotic bodies. - Induced protein expression of cytochrome c, active caspase-3, and caspase-9. - DNA damage.	[177]
	SRM® 1648a	50–200 ppm, systemic	24 and 48 h	- Increased ROS production.	[130]
	SRM® 1648a SRM® 1649b	50 μg/cm^2^, systemic	24 h	- Nuclear translocation of AhR. - Increased *CYP1A1* and *CYP1B1* mRNA expression.	[178]
	SRM® 1649b	50 μg/cm^2^, systemic	1 h	- Increased levels of intracellular ROS.	[142]
	SRM® 1649b	50 μg/cm^2^, systemic	2 and 24 h	- Increased cellular and mitochondrial ROS levels after 2 h. - Increased HMOX1 protein expression after 24 h.	[143]
	SRM® 1649b	25 and 50 μg/cm^2^, systemic	4 and 24 h	- Increased ROS production. - Increased NOX activity.	[122]
	SRM® 1649b	25–100 μg/cm^2^, systemic	4 and 24 h	- Increased NOX2 protein expression. - Increased ROS production.	[179]
	SRM® 1649b	25 μg/mL, systemic	2 and 24 h	- Increased ROS production.	[180]
	SRM® 1649b	50 μg/cm^2^, systemic	1 and 4 h	- Increased ROS production. - Increased NOX2 activity.	[181]
	SRM® 1649b	25 and 50 μg/cm^2^, systemic	4 and 24 h	- Increased ROS production - Increased NOX activity.	[122]
	SRM® 1649b	100–400 μg/mL, systemic	24 h	- Nuclear translocation of AhR. - Increased *CYP1A1* mRNA expression. - Induced apoptosis.	[172]
	SRM® 1650b	50 μg/mL, systemic	24 h	- Increased cellular and mitochondrial ROS levels and mitochondrial stress. - Increased lipid peroxidation and protein carbonylation. - Increased cleaved caspase-3 and BAX and decreased Bcl-2 protein expression. - Induced DNA damage. - Increased number of apoptotic bodies.	[182]
	SRM® 1650b	50 μg/mL, systemic	1 and 24 h	- Increased intracellular ROS and superoxide anion levels. - Increased levels of protein carbonylation and lipid peroxidation after 24 h. - Induced DNA damage and apoptotic body formation. - Increased mitochondrial permeability after 24 h. - Increased BAX, active caspase-3, and PARP and decreased Bcl-2 protein expression.	[183]
	SRM® 1650b	50 μg/mL, systemic	24 h	- Increased intracellular ROS and calcium levels. - Increased levels of lipid peroxidation and protein carbonylation. - DNA damage. - Increased mitochondrial ROS, calcium, and permeability. - Apoptosis: increased protein expression of ATF6, GRP78, p-IRE1, BAX, and active caspase-3 and caspase-9. Decreased protein expression of Bcl2. Increased number of apoptotic bodies. - Autophagy: autophagic lysosomes. Increased protein expression of LC3B-II and beclin-1.	[168]
	SRM® 1650b	50 μg/mL, systemic	24 h	- Increased intracellular ROS and superoxide levels. - Induced NOX activity. - Increased intracellular calcium levels and mitochondrial membrane permeability. - Induced lipid peroxidation and protein carbonylation. - DNA damage. - Increased number of apoptotic bodies.	[184]
	SRM® 1650b	50 μg/mL, systemic	1, 4, 8, 12, and 24 h	- Increased intracellular ROS. - Increased levels of intracellular calcium. - ER stress: Increased protein expression of CHOP, GRP78, and p-PERK. - Increased mitochondrial permeability. - DNA damage. - Increased lipid peroxidation and protein carbonylation. - Apoptosis: Increased protein expression of BAX, DNA breakage, apoptotic body formation, and increased expression of active caspase-3 and caspase-9. - Autophagy: Increased protein expression of LC3B-II.	[123]
	SRM® 1650b	50 μg/mL, systemic	30 m, 1 h, and 24 h	- Increased ROS production. - Increased levels of intracellular calcium. - Induced senescence.	[185]
	SRM® 1650b	50 μg/mL, systemic	24 h	- Increased ROS production. - Increased lipid peroxidation. - Increased number of apoptotic bodies.	[186]
	SRM® 1650b and 2975	10 and 100 μg/mL, systemic	1 and 24 h	- No changes in radical production.	[174]
	SRM® 2975	100 and 200 μg/mL, systemic	24 h	- Increased protein expression of cleaved caspase-3 and PARP. - Increased protein expression of BAX and p53.	[125]
	CRM no. 28	125 μg/mL, systemic	30 m, 24 h post-incubation	- Increased levels of intracellular ROS.	[137]
	ERM-CZ120	100 μg/mL, systemic	30 m	- Increased intracellular ROS production.	[126]
	ERM-CZ120	25–100 μg/mL, systemic	3 and 24 h	- Increased CYP1A1 protein expression. - Decreased AhR protein expression. - Increased LC3-II and p62 protein expression.	[187]
	CAPs, PM_2.5_	5–25 μg/mL, systemic	1, 3, 6, and 24 h	- Increased HNE protein adduct formation. - Increased nuclear translocation of Nrf2. - No changes in *GPX*, *GR*, and *NPQO1* mRNA expression.	[145]

Abbreviations: h, hour; d, day; m, minute; HSE, human skin equivalent; HEE, human epidermal equivalent; NHEK, normal human epidermal keratinocyte; SRM, standard reference material; PM, particulate matter; CAP, concentrated ambient particles; ppm, parts per million; CRM, certified reference material; ROS, reactive oxygen species; PARP, poly (ADP-ribose) polymerase; Nrf2, nuclear factor erythroid 2-related factor 2; AhR, aryl hydrocarbon receptor; LC3-II, light-chain 3 II; BIRC5, baculoviral IAP repeat containing 5; NQO1, NAD(P) H quinone dehydrogenase 1; HMOX1, heme oxygenase 1; NOX, NADPH oxygenase; HNE, 4-hydroxy-2-nonenal; ER, endoplasmic reticulum; CYP1A1, cytochrome P450 family 1 subfamily A member 1

#### PM triggers exogenous and endogenous ROS formation

PM can trigger the formation of exogenous ROS levels through the formation of free radicals as a result of the high particle surface reactivity [188, 189]. Diesel PM and its organic extract were shown to both possess high reactivity by generating ROS via the catalyzation of oxygen reduction by various reductants [190]. In addition, PAHs and quinones that are bound to the PM’s surface have shown strong redox activity in vitro [191]. Transition metals (e.g., copper and iron) present in PM can generate ROS through Fenton reactions [192]. In keratinocytes, redox-active components such as copper and quinones were shown to induce oxidative stress by means of inducing the nuclear translocation of a redox-sensitive protein complex, causing mitochondrial stress and finally leading to apoptosis [193]. The redox activity of PM is correlated to the size and the adsorbed species on the surface [194]. Higher redox activity per PM mass was measured for ultrafine particles, compared to particles of a larger size. Moreover, a strong correlation was found between PM’s redox activity and the mass fractions of species they carry (i.e., metals and PAHs).

Indirectly, PM or its adsorbed species (e.g., PAHs, quinones, metals) can increase endogenous ROS generation by inducing mitochondrial stress and increasing the activity of ROS producing enzymes. The mitochondria are commonly considered as the main source of ROS production in the cell. PM induces mitochondrial dysfunction, by inducing major structural damage [123, 135, 195]. Typically, mitochondrial dysfunction is indicated by reduced ATP levels, as determined in PM_2.5_-treated keratinocytes and fibroblasts [175]. This dysfunction leads to the generation of mitochondrial ROS, and to increased radical production and mitochondrial Ca^2+^ levels in vitro [123, 143, 174, 175, 182]. As a result, the intracellular ROS levels were shown to increase substantially after 30 min of PM-treatment up to 24 h (e.g., 50 μg/mL) in 2D skin models [119, 121–124, 126, 130, 137, 138, 142, 143, 168, 171, 173, 175, 176, 179–186].

The endoplasmic reticulum (ER) is responsible for most of the intracellular Ca^2+,^ and, as a result, changes in the cellular Ca^2+^-balance activate ER stress [196]. PM was shown to induce ER stress, as measured by increased levels of intracellular Ca^2+^ [123, 168, 175, 184–186]. Additionally, the ER stress sensor proteins for unfolded or misfolded proteins, protein kinase-like ER kinase (PERK), inositol-requiring 1 alpha (IRE1α), and activating transcription factor 6 (ATF6) [197], were activated in vitro [123, 175]. In vivo, mitochondrial and ER stress was observed after longer (e.g., 7 days) topical PM-exposure, as indicated by mitochondrial and ER swelling [123].

Sensing exogenous chemical substances is paramount to stimulating an immune response as the first line of defense against these compounds. An important sensor of environmental chemicals is the aryl hydrocarbon receptor (AhR), a transcription factor that is highly expressed in all skin cells [198]. Besides the xenobiotic metabolism and preserving the skin’s homeostasis, the AhR plays a role in epidermal differentiation and barrier function of the skin [199, 200]. Common synthetic exogenous ligands are PAHs and halogenated aromatic hydrocarbons (HAHs) such as 2,3,7,8-tetrachlorodibenzodioxin (TCDD), dimethylbenz [a] anthracene (DMBA), methylcholanthrene or benzo[a]pyrene (BaP), coming from environmental air pollutants, such as PM [201]. Upon binding to its ligand, the AhR translocates from the cytoplasm to the nucleus, where it forms a dimer with the AhR nuclear translocator (ARNT) [202]. PM induces the nuclear translocation of AhR in vitro [119, 124, 171, 172, 178]. The AhR/ARNT complex binds to conserved promoter regions containing the xenobiotic response element (XRE), promoting the transcription of several groups of target genes, such as from the phase I metabolism (e.g., cytochrome P450 family 1 subfamily A member 1, *CYP1A1*, *CYP1A2,* and *CYP1B1*), the phase II metabolism (e.g., UDP glucuronosyltransferase family 1 member A complex locus, *UGT1A* and glutathione S-transferase A1, *GSTA1*) and a gene for the aryl-hydrocarbon receptor repressor (*AhRR*) [203]. CYP enzymes can metabolize PAHs, and the formed metabolites can induce cell damage either by the formation of DNA, protein adducts, or the generation of ROS [195, 204–208]. Indeed, PM upregulates the CYP enzyme mRNA and protein expression in vitro and in vivo [119, 128, 129, 134, 135, 139, 141, 167, 171, 172, 178, 187].

Other ROS producing enzymes are the members of the nicotinamide adenine dinucleotide phosphate (NADPH) oxidase (NOX) family [209]. Once activated by PM [122, 169, 173, 179, 181, 184], these enzymes generate high levels of ROS.

#### PM-induced oxidative stress results in lipid, protein, and DNA damage

The skin is covered by a thin layer of sebum, that functions as a barrier against water evaporation (i.e., TEWL) [210]. Sebum lipids, such as squalene, wax, cholesterol (esters), triglycerides, and free fatty acids, are a target for peroxidation by ROS. In addition to oxidation by exogenous ROS, endogenous ROS can target polyunsaturated fatty acids (PUFAs) from cell membranes, leading to the generation of reactive aldehyde byproducts like malondialdehyde (MDA) and 4-hydroxy-2-nonenal (HNE) [211], commonly used as biomarkers for oxidative stress [212, 213]. Skin exposure to PM was found to induce lipid peroxidation, as well as elevated levels of reactive aldehyde byproducts [119, 123, 128, 133, 145, 168, 175, 177, 182–184]. These reactive aldehydes can, in turn, react with amino acid residues in proteins, resulting in the formation of carbonylated proteins [214], which were also detected in the skin upon treatment with PM [123, 168–171, 175, 182–184]. The introduction of carbonyl groups (e.g., aldehydes and ketones) in proteins can also be caused by oxidative cleavage or the direct oxidation of amino acid residues [214]. The formation of these highly stable carbonyl groups results in conformational changes and irreversible damage to the polypeptide chain. Subsequent unfolding and complete inactivation of the protein allows for protein crosslinking, leading to the formation of adducts [215]. Also, DNA can be a target for ROS, and PM was shown to induce the formation of DNA adducts in mice skin [166].

#### Counteracting PM-induced oxidative stress

The first line of defense against ROS is the cornified envelope, especially the SPRR family [216]. It was shown that cysteine residues within the SPRR proteins are responsible for ROS quenching, most likely due to its location on the outer layer of the skin [217]. Additionally, it was shown that common skin bacteria secrete specific proteins to counteract oxidative stress [218].

Other defense mechanisms, to abrogate oxidative stress processes, are identified like the upregulation of antioxidant proteins and detoxifying enzymes that are activated by transcription factors via antioxidant response elements (ARE) [165]. The expression and the subsequent nuclear translocation of one of these transcription factors, namely the nuclear factor erythroid 2-related factor 2 (Nrf2), is increased in vitro upon exposure to PM [127, 138, 145]. In turn, Nrf2 target genes and their protein expression such as heme oxygenase 1 (HMOX1), NAD(P) H quinone dehydrogenase 1 (NQO1), glutathione S-transferases (GSTs) are upregulated [127, 133, 138, 141, 143].

Activation of the AhR induces the nuclear translocation of Nrf2 by a mechanism that needs to be elucidated, but evidence suggests it involves protein kinases that induce transcription of detoxifying enzymes, leading to a decrease of ROS levels [219].

#### PM-induced apoptosis and autophagy

Oxidative stress can lead to the activation of programmed cell death, i.e., apoptosis [220]. Protein levels of CCAAT-enhancer-binding protein homologous protein (CHOP), a transcription factor that mediates ER-stress induced apoptosis [221] and endoplasmic reticulum chaperone BiP (GRP78), a key element in normal ER function [222], were determined to be upregulated by PM_2.5_ [123, 168, 175]. Mitochondrial dysfunction leading to mitochondria-dependent apoptosis is characterized by increased permeability of the mitochondrial membrane [223]. Mitochondria-dependent apoptosis (e.g., intrinsic pathway of apoptosis) is mediated by the release of cytochrome c and caspase activation (e.g., caspase-3 and caspase-9) [223]. It was shown that PM induces mitochondrial-dependent apoptosis, indicated by an increased permeability of the mitochondrial membrane [123, 168, 175, 177, 183, 184], increased cellular cytochrome c [177], and activation of caspase-3, caspase-9 and poly (ADP-ribose) polymerase (PARP) [123, 125, 133, 168, 175, 177, 182–184, 186]. Furthermore, PM-induced apoptosis was observed by and upregulation of apoptosis regulator BAX, a downregulation of anti-apoptotic proteins Bcl-2 and Mcl-1 [123, 168, 175, 182], DNA fragmentation [123, 128, 168, 175, 177, 182–184], and the formation of apoptotic bodies [123, 137, 168, 172, 175, 177, 182–184, 186].

ER stress is also related to autophagy, and PM exposure was shown to promote autophagy in vitro*.* Proteins involved in the initiation of autophagosome formation, such as light-chain 3B II (LC3B-II) and Beclin 1, were shown to be upregulated [123, 135, 168, 175, 224].

#### PM-induced senescence

Another mechanism that can be activated upon ROS-induced DNA damage is cellular senescence. Senescent cells are in irreversible arrest, thereby limiting proliferation, that is implicated in skin aging [225]. It was shown that PM_2.5_ induces senescence in human keratinocytes (i.e., NHEK, HaCaT, and HEK001) [124, 172, 185]. PM-induced senescence was shown to be dependent on ROS formation and subsequent AhR activation [124]. The senescence inducer protein p16^INK4A^ was found to be upregulated upon PM_2.5_ treatment, and its transcription was shown to be epigenetically regulated through promoter methylation [124]. In mice, topical PM exposure led to a hyperkeratotic epidermis. Epidermal hyperproliferation might result from the activation of the AhR, which is known to induce increased keratinocyte proliferation and differentiation [200].

### Activation of the inflammatory cascade

The skin plays a major role in protecting the body from harmful environmental factors. The keratinocytes and immune cells contribute to the first and second lines of defense of the skin’s immune system and are an indispensable part of the immunological barrier compartment. Additionally, the dermal fibroblasts play an important role in the sensing of pathogens, to protect the body further from harmful intrusive species. The reported findings on the effects of PM on the activation of the inflammatory cascade in skin models have been summarized (Table 3).

**Table 3:** Review of reported effects on inflammatory cascade in varying skin models upon exposure to PM

Model	PM type	Dose and application	Exposure time	Main findings	Ref.
Ex vivo human skin	SRM® 1649b	2 mg/cm^2^, topical	24 h	- Increased MMP-1 protein expression.	[119]
Mice, BALB/c	SRM® 1649b	100 μg/m^2^, topical	24 h repetitive exposure, 5 d total	- Increased COX2 protein expression.	[122]
	SRM® 1649b	100 μg/cm^2^, topical	24 h repetitive exposure, 5 d total	- Increased epidermal thickness. - Neutrophil infiltration. - Increased COX2 protein expression.	[179]
Mice, BALB/c with disrupted barrier	PM ≤1 μm from Seoul, Korea	8 μg/cm^2^, topical	6 h and 2 w repetitive exposures, 10 times in total	- Increased epidermal thickness in both skin types. - Neutrophil infiltration. - Increased mRNA expression of IL-8 functional homologs (*KC*, *MIP2*, *LIX*). - Increased mRNA expression *MMP13*.	[121]
Mice, NC/Nga, AE model	The soluble phase of DEP.	1 mg/time, topical	Repetitive exposure, every 1, 3, or 9 weeks in total	- Increased AE lesion formation. - Increased total IgE levels.	[226]
Mice, HR-1	SRM® 1650b	100 μg/mL, topical	7 d	- Increased epidermal thickness.	[124]
	SRM® 1650b	100 μg/mL, topical	7 d	- Increased epidermal thickness.	[168]
	SRM® 1650b	100 μg/mL, topical	7 d	- Increased IL-1β and IL-6 protein expression. - Increased TLR5 protein expression. - Increased MyD88 protein expression. - Increased phosphorylation of p65.	[169]
HSE	SRM® 2975	200 μg/mL, systemic	Every 2 d, 6 d total	- Decreased IL-8, CXCL10, and ICAM1 protein secretion.	[125]
HEE	CAPs, PM_2.5_	0.5 and 2.0 μg/cm^2^, topical	24 and 48 h	- Nuclear translocation of p65. - Increased IL-1α protein secretion. - Increased COX2 protein expression.	[128]
	CRM no. 28	25 mg, topical	6 and 24 h	- Upregulated mRNA expression of *IL1A*, *IL1B*, *IL6*, *CXCL8*, *CCL20*, *MMP1*, *MMP3*, *MMP9*, *MMP12*, and *ICAM1* after 6 h. - Downregulated expression of tissue inhibitors of MMPs (2–4) after 6 h. - Induced IL-8 and MMP-1 protein secretion after 24 h.	[129]
	PM_0.3–2.5_ from Benin, West-Africa	15 and 30 μg/cm^2^, topical	24 h	- Increased IL-1α and IL-8 protein secretion. - Increased MMP-1 and MMP-3 protein expression.	[133]
	PM_2.5_ from Seoul, Korea	50 μg/mL, topical	24 h	- Increased MMP-1 protein expression.	[132]
	SRM® 1648a	2.2, 8.9, and 17.9 μg/cm^2^, topical	24 and 48 h	- Increased IL-1α protein secretion.	[131]
NHDF	SRM® 1649b	100–400 μg/mL, systemic	24 h	- Phosphorylation of ERK and JNK. - Increased *MMP1* mRNA and protein expression.	[172]
	Diesel PM	30 and 60 μg/mL, systemic	24 h	- Increased *MMP3* and *MMP9* mRNA expression.	[138]
	SRM® 1649b	50 μg/mL, systemic	24 h	- Increased *MMP1* mRNA expression.	[119]
	SRM® 2787	30 μg/cm^2^, systemic	24 h	- Increased IL-6 and IL-8 protein secretion. - Increased MMP-1 and decreased procollagen and TGF-β protein secretion. - Increased *IL1B*, *IL6*, *CXCL8*, and *IL33* mRNA expression. - Increased *MMP1* and *MMP3* and decreased *TGFB*, *collagen type I alpha 1 chain*, *collagen type I alpha 2 chain*, and *elastin* mRNA expression.	[135]
	ERM-CZ100	200 μg/mL, systemic	24 h	- Increased MMP-1, −2,-8, −9, −13 protein expression. - Nuclear translocation of p65. - Phosphorylation of ERK, JNK, and p38.	[136]
	The pre-conditioned medium of HaCaT treated with CRM no. 28	125 μg/mL, systemic	30 m, 48 h post-incubation	- Increased PGE_2_, TNF-α, IL-1β, and IL-6 protein secretion. - Increased COX2 protein expression. - Nuclear translocation of p65. - Phosphorylation of p38, ERK, and JNK. - Increased MMP-1 and MMP-2 protein expression.	[137]
	Diesel PM	30 and 60 μg/mL, systemic	6 h	- Increased *MMP2* and *MMP9* mRNA expression.	[227]
NHDF and HaCaT co-culture	SRM® 1648a and SRM® 1649b	50 μg/cm^2^, systemic	24 h	- Phosphorylation of p38. - Increased *IL1A*, *IL1B*, *IL6*, and *CXCL8* mRNA expression and protein secretion (HaCaT). - No changes in *TNF* mRNA expression and protein secretion (HaCaT). - Increased *MMP1* and *COX2* mRNA expression (NHDF).	[178]
NHEK	PM_2.5_ from Xi’an, China	50 μg/mL, systemic	24 h	- Top upregulated genes from transcriptomics analysis are *CXCL1*, *IL1A*, and *IL1B*.	[134]
	Diesel PM	30 and 60 μg/mL, systemic	24 h	- Increased *IL1A*, *IL6*, *CXCL8*, and *TNF* mRNA expression and protein secretion.	[138]
	ERM-CZ120	3, 10, 30 and 100 μg/mL, systemic	24 and 48 h	- Increased *IL1B*, *IL6*, *CXCL8*, and *TNF* mRNA expression after 24 h. - Increased IL-1β, IL-6, IL-8, and TNF-α protein secretion after 48 h. - Increased *MMP1* mRNA expression and protein secretion after 24 h.	[173]
	SRM® 1650b	Unknown	24 h	- Increased IL-1β and IL6 protein secretion. - Increased IL-6 protein expression.	[169]
	PM ≤1 μm from Seoul, Korea	40 μg/cm^2^, systemic	24 h	- Increased *CXCL8* mRNA expression and protein secretion. - Increased *MMP1* mRNA expression and protein secretion.	[121]
	SRM® 2786	1 mg/mL, systemic	6 h	- RNA-Seq analysis: Upregulation of *IL1B*, *IL36G*, *CXCL3*, *CXCL8*, and *IL1R2*. Downregulation of *MMP3* and *MMP28*.	[141]
	SRM® 1649b	50 μg/cm^2^, systemic	24 h	- Increased COX2 protein expression.	[181]
	SRM® 1649b	100 μg/mL, systemic	24 and 48 h	- Increased IL-8 protein secretion.	[228]
	Diesel PM	20 μg/mL, systemic	48 h	- Increased IL-1β and IL-8 protein secretion.	[229]
	Diesel PM	30 and 60 μg/mL, systemic	6 h	- Increased *IL1A*, *IL6*, *CXCL8*, and *TNF* mRNA expression.	[227]
	Asian dust storm particles from Seoul, Korea	25 μg/mL, systemic	24 h	- Increased *IL6*, *CXCL8*, and *CSF2* mRNA expression. - No changes in *IL1B*, *IFNG*, and *IL18* mRNA expression.	[139]
	PM_2.5_ from Seoul, Korea	25 μg/mL, systemic	24 h	- Top upregulated genes from transcriptomics analysis are *IL1B*, *IL36G*, *IL1A*, *IL1R2*, *PTGS2*, *IRAK2*, *MMP1*, *MMP9*, and *MMP10*. - Downregulated gene is *CXCL14*. - Induced IL-1α protein secretion. - Phosphorylation of p65 and p38.	[132]
HaCaT	CRM no. 28	125 μg/mL, systemic	30 m, 48 h post-incubation	- Increased PGE_2_, TNF-α, IL-1β, and IL6 protein secretion. - Increased COX2 protein expression. - Nuclear translocation of p65. - Phosphorylation of p38, ERK, and JNK. - Increased MMP-1 and MMP-2 protein expression.	[137]
	PM_2.5_ from Shanghai, China	10–100 μg/mL, systemic	24 h	- No changes in CSF2 protein secretion. - Increased TSLP, TNF-α, IL-1α, and IL-8 protein secretion.	[144]
	SRM® 1648a	50–200 ppm, systemic	24 and 48 h	- Increased *TRPV1* mRNA and protein expression (via p38/MAPK and NF-κB pathway). - Increased IL-1β, IL-8, and TNF-α protein secretion.	[130]
	SRM® 1648a and SRM® 1649b	50 μg/cm^2^, systemic	24 h	- Induced phosphorylation of p-38.	[178]
	SRM® 1649b	50 μg/cm^2^, systemic	6 and 24 h	- Increased phosphorylation of ERK, p38, JNK, and Akt after 6 h. - Increased COX2, ICAM1, cPLA_2_, and PGE_2_ protein expression after 24 h. - Increased MMP-9 protein expression after 24 h.	[143]
	SRM® 1649b	50 μg/cm^2^, systemic	2 and 6 h	- Increased COX2 and MMP-9 protein expression. - Phosphorylation of ERK, JNK, and p38.	[142]
	SRM® 1649b	25 and 50 μg/cm^2^, systemic	4 and 24 h	- Increased COX2 protein expression. - Increased PGE_2_ and IL-6 protein secretion. - No changes in IL-24, IL-1β, and TNF-α protein secretion.	[122]
	SRM® 1649b	25–100 μg/cm^2^, systemic	4 and 24 h	- Increased COX2 protein (24 h) and mRNA (6 h) expression. - Increased PGE_2_ protein secretion after 24 h. - Nuclear translocation p65 after 4 h. - Phosphorylation of ERK, JNK, and p38 after 4 h.	[179]
	SRM® 1649b	25 μg/mL, systemic	2 and 24 h	- Increased IL-6, IL-1β, and IL-1α protein secretion, and mRNA expression. - Phosphorylation of p38 and AP-1.	[180]
	SRM® 1649b	50 μg/mL, systemic	UK	- Increased protein expression of COX2, PLA_2_, ICAM1, and MMP-9. - Phosphorylation of ERK, p38, and JNK.	[230]
	SRM® 1649b	50 μg/cm^2^, systemic	6 and 24 h	- Increased COX2 protein (24 h) and mRNA (6 h) expression. - Increased PGE_2_ protein secretion. - Phosphorylation of p38, ERK, JNK, and p65.	[181]
	SRM® 1649b	100–400 μg/mL, systemic	24 h	- Phosphorylation of ERK and JNK. - Increased *MMP1* mRNA and protein expression.	[172]
	SRM® 1650b	Unknown	1 h	- Binding of PM to TLR5.	[169]
	SRM® 1650b	50 μg/mL, systemic	24 h	- Induced phosphorylation of ERK, p38, and JNK.	[182]
	SRM® 1650b	Unknown	3 h	- Increased IL-1β, IL-6, IL-8, IL-16, and TGF-β2 protein secretion. - Increased *IL6* mRNA expression.	[169]
	SRM® 1650b	Unknown	24–72 h	- Increased IL-1β, IL-6, IL-8, IL-16, and TGF-β2 protein secretion. - Increased IL-6 protein expression (time-dependent increase). - Increased *IL6* mRNA expression. - Increased TLR5 protein and mRNA expression. - Increased MyD88 protein expression. - Nuclear translocation of p65. - NOX4-TLR5 interaction.	[169]
	SRM® 1650b	50 μg/mL, systemic	24 h	- Phosphorylation of ERK, JNK, and p38.	[168]
	SRM® 1650b	50 μg/mL, systemic	30 m, 1 h, and 24 h	- Increased MMP-1 protein activity, expression, and mRNA expression. - Increased MMP-2 and MMP-9 protein expression. - Activation of the MAPK pathway. - AP-1 binding to MMP-1 promotor.	[185]
	SRM® 2975	100 and 200 μg/mL, systemic	30 m and 24 h	- Decreased IL-8 and CXCL10 protein secretion after 24 h. - Phosphorylation of p38, ERK, and JNK after 30 min.	[125]
	CAPs, PM_2.5_	5–25 μg/mL, systemic	1, 3, 6 and 24 h	- Nuclear translocation of p65. - Increased IL-1α protein secretion.	[145]
	CRM no. 28	125 μg/mL, systemic	30 m, 24 h post-incubation	- Nuclear translocation of p65. - Phosphorylation of p38, ERK, and JNK. - Increased PGE_2_, TNF-α, and IL-6 protein secretion. - Increased COX2 protein expression.	[137]
	CRM no. 28	125 μg/mL, systemic	30 m, 24 h post-incubation	- Nuclear translocation of p65. - Phosphorylation of p38. - Increased COX2 protein expression. - Increased PGE_2_, IL-6, TNF-α, and IL-1β protein secretion.	[231]
	ERM-CZ100 and ERM-CZ120	100 μg/mL, systemic	24 h	- Increased IL-1β, PGE_2_, and IL-6 protein secretion. - Increased COX2 protein expression.	[232]
	ERM-CZ120	100–400 μg/mL, systemic	24 and 48 h	- Increased PGE_2_ protein secretion.	[126]
HEK-001	SRM® 1650b	Unknown	24 h	- Increased IL-1β and IL-6 protein secretion. - Increased IL-6 protein expression.	[169]
JB6 P+, mouse cell line	SRM® 1975	10–20 μg/mL, systemic	12, 24, 36, and 48 h	- Activation of NF-κB. - No activation of AP-1. - Activation of the Akt/PI3K pathway. - Phosphorylation of ERK, not of p38 and JNK.	[233]

Abbreviations: h, hour; d, day; HSE, human skin equivalent; HEE, human epidermal equivalent; NHEK, normal human epidermal keratinocyte; SRM, standard reference material; PM, particulate matter; CAP, concentrated ambient particles; ppm, parts per million; CRM, certified reference material; CXCL10, C-X-C motif chemokine ligand 10; TLR, Toll-like receptor; IL, interleukin; MAPK, mitogen-activated protein kinase; TNF, tumor necrosis factor; MMP, matrix metalloprotease; NF-κB, nuclear factor kappa B; PGE_2_, prostaglandin E2; AP-1, adaptor protein 1

### Activation of toll-like receptors

Keratinocytes in the viable epidermis (i.e., SG, SS, and SB) and dermal fibroblasts defend the human body by sensing pathogens and mediating an immune response by differentiating between harmful and harmless pathogens [28]. They do so by the recognition of pathogen-associated molecular patterns (PAMPs), which are evolutionary conserved small molecular motifs within microbes, including lipopolysaccharide (LPS), peptidoglycan, flagellin and nucleic acids [234]. These PAMPs trigger responses once they are recognized by pattern recognition receptors (PRR) [235], such as members of the Toll-like receptor (TLR) family, located at the cell surface or in the cytoplasm [236]. Keratinocytes constitutively express TLR1–6 and TLR9 [237] whereas fibroblasts express all TLRs (i.e., 1–10) [238]. Their activation is critical for provoking distinct immune responses in the skin [236].

Activated TLRs can recruit and activate the adaptor molecule, myeloid differentiation primary response 88 (MyD88), to initiate signaling and activate the nuclear factor kappa B (NF-κB) pathway and members of the mitogen-activated protein kinase (MAPK) family [237, 239, 240].

#### PM-induced activation of NF-κB

NF-κB represents a group of five inducible transcription factors that, once activated, mediate transcription of target genes by binding to a specific DNA element. Its target genes include an enormous amount of chemokines and cytokines among which tumor necrosis factor (*TNF*), interleukin 1 alpha (*IL1A*), interleukin 1 beta (*IL1B*) and chemokines such as C-X-C motif chemokine ligand 8 (*CXCL8*), have been shown to play a key role in the skin [241]. In turn, the tumor necrosis factor (TNF), interleukin 1-alpha (IL-1α), and interleukin 1-beta (IL-1β) proteins can bind their respective receptors and transactivate NF-κB. Other target genes encode adhesion molecules like intercellular adhesion molecule 1 (*ICAM1*) and enzymes such as cyclooxygenase 2 (*COX2*) and inducible nitric oxide synthase (*iNOS*) [241]. COX2 is an enzyme that is crucial for the formation of prostaglandin E_2_ (PGE_2_) derived from arachidonic acid from membrane phospholipids [242].

PM_2.5_ was shown to be able to bind to TLR5 in keratinocytes [169]. Additionally, it was determined that TLR5 interacts with MyD88 following PM exposure, both in vivo (mice) and in vitro [169]. This, in turn, initiates the signaling cascade, and upon activation of NF-κB, the p65 subunit translocates to the nucleus, where it regulates the expression of its target genes that are involved in various processes of the inflammatory response [241]. Indeed, PM induces the phosphorylation and nuclear translation of the p65 unit [128, 130, 136, 137, 145, 169, 179, 181, 231, 233]. The PM-induced activation of NF-κB leads to the increased transcription of its target genes and their protein expressions such as IL-1α, IL-1β, IL-6, IL-8, ICAM1, COX2 and TNF-α [124, 128, 130, 133, 135, 138, 139, 142, 169, 173, 181, 227, 229, 230, 243]. PM-induced COX2 expression leads to induced production of PGE_2_ [122, 126, 137, 143, 179, 181, 231, 232].

Activation of NF- κB also leads to the transcription of the NLR family pyrin domain containing 3 (*NLRP3*) gene, which is a crucial priming signal for the activation of the inflammasome [241]. The inflammasome is a large multi-protein, and its assembly leads to the activation of caspase-1, which in turn results in the cleavage of pro-IL-1β and pro-IL-18 that are stored in keratinocytes into biologically active IL-1β and IL-18, respectively [244–246]. A subsequent release enables adjacent epithelial cells to amplify this signal by stimulating the production of IL-1α, TNF-α, and IL-6. PM-induced activation of the inflammasome in the skin has not yet been studied; however in the pulmonary [247] and cardiovascular [248] systems, it was shown that PM activates the NLPR3 inflammasome in vivo (e.g., in mice).

Secretion of these chemokines and cytokines results in the regulation of an immune response by recruiting and activating different immune cell types into the skin, such as memory and effector T cells, and Langerhans cell precursors into the dermis and epidermis [249–251]. In vivo, the activation of the inflammatory cascade was observed through activation of NF-κB signaling with a resulting increased expression of pro-inflammatory cytokines upon topical exposure to PM [121, 122, 169]. Additionally, an increase in epidermal thickness (i.e., an indicator for activation of the inflammatory cascade) and neutrophil infiltration was observed [121, 168, 179, 226].

In animal models with a disrupted skin barrier, topical PM application resulted in an upregulated expression of the functional homolog of IL-8, dermal cell infiltration, and increased epidermal thickness [121]. This indicates that PM can aggravate symptoms from skin diseases with an already existing disrupted skin barrier like AE, supporting the epidemiological evidence that airborne PM can aggravate AE symptoms. Moreover, in AE mice models, topical application with the soluble phase of diesel PM (e.g., PAHs), resulted in increased lesion formation and increased immunoglobulin E (IgE) levels [226].

#### PM induces activation of MAPK proteins

A different adaptor molecule, called TIR-domain-containing adapter-inducing interferon-β (TRIF), can be recruited by TLRs (e.g., TLR 3 and 4) and, in addition to MyD88, can initiate signaling of both NF-κB and MAPK [240]. This activation of downstream signaling pathways results in the activation of MAPK members, such as extracellular-signal-regulated kinase (ERK), c-Jun-NH2-terminal kinase (JNK), p38, but also the transcription factor adaptor protein 1 (AP-1) [252]. Exposure of fibroblasts and keratinocytes to PM was shown to induce the activation of the MAPK pathway by the phosphorylation of ERK, JNK, and p38 [130, 136, 143, 168, 172, 178, 179, 181, 184].

A crosstalk between the previously described AhR and the MAPK pathway has been reported [253]. The proto-oncogene tyrosine-protein kinase (c-Src), which is part of the AhR complex, is activated and released upon the binding of the AhR to its ligand [253]. The soluble c-Src can in turn bind the epidermal growth factor receptor (EGFR) and activate the MAPK pathway [253].

MAPK activation and the resulting activation of AP-1 leads to transcriptional regulation of matrix metalloproteinases (MMPs). The major sources of MMPs in the skin are keratinocytes and fibroblasts [254], and they are responsible for remodeling of the dermis by the controlled degradation of the extracellular matrix (i.e., fragmentation of collagen fibrils) [255]. As a result, higher MMP expression and activity leads to accelerated skin aging [256]. Increased gene and protein levels of several matrix metalloproteinases (MMPs) were determined upon PM exposure in vivo and in vitro [119, 121, 129, 132, 133, 135, 137, 138, 141–143, 172, 173, 178, 185, 227]*.*

### Working model of the main affected pathways upon PM exposure to skin

**Fig. 2:**
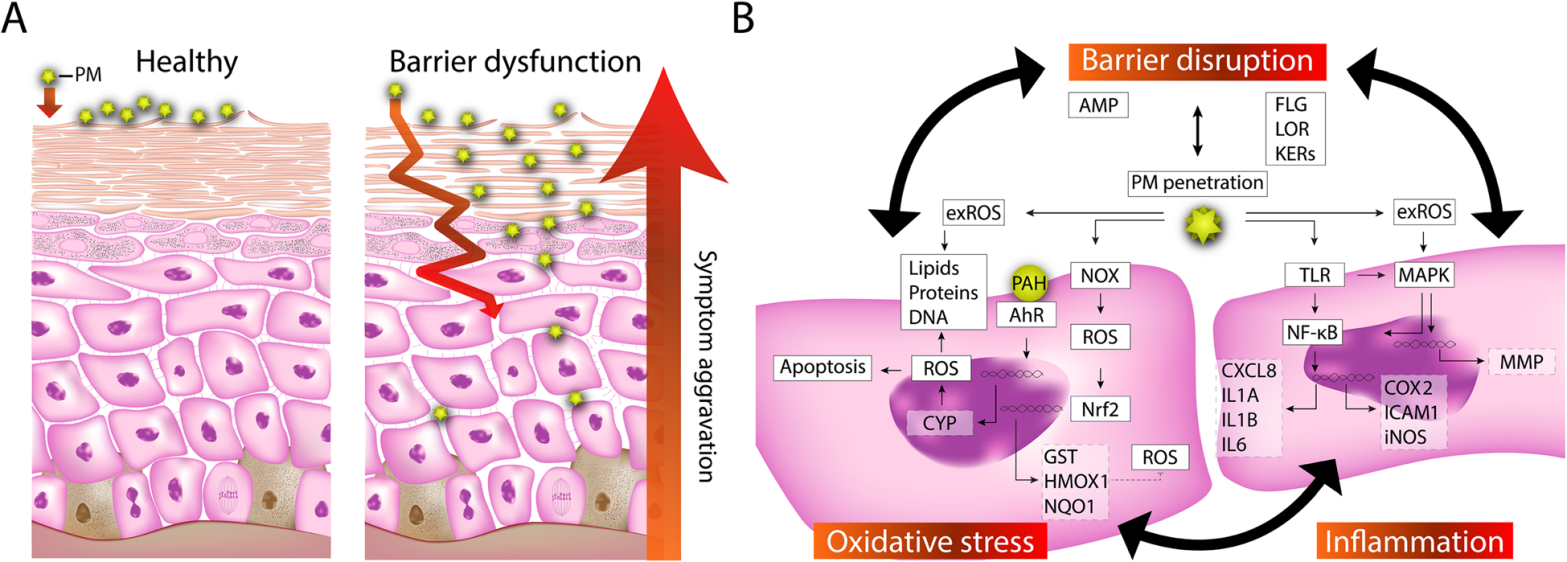
The main affected pathways upon exposure of the skin to airborne PM. **(A)** PM disrupts the barrier of skin with an already existing barrier dysfunction to a greater extent. **(B)** The underlying mechanism of the effects of PM exposure on the skin. PM can disrupt the epidermal barrier by increasing the levels of antimicrobial peptides (AMP) and inhibiting the levels of proteins that are essential for cell differentiation and proliferation (i.e., FLG: Filaggrin, LOR: Loricrin, KERs: Keratins). PM induces both exogenous ROS (exROS) and endogenous ROS (ROS) formation by activation of the aryl hydrocarbon receptor (AhR), upon exposure to polycyclic hydrocarbons (PAHs), and increased activity of the nicotinamide adenine dinucleotide phosphate (NADPH) oxidase enzymes (NOX). A misbalance of ROS and antioxidant scavengers results in oxidative stress. ROS activates the nuclear translocation of nuclear factor erythroid 2-related factor 2 (Nrf2) to propagate oxidative stress, by inducing the gene transcription of glutathione S-transferase (*GST*), heme oxygenase 1 (*HMOX1*), and NAD(P) H quinone dehydrogenase 1 (*NQO1*). ROS can cause damage to the skin by lipid peroxidation, protein carbonylation, and can cause irreversible cellular impairment through DNA damage. These damages, together with mitochondrial dysfunction, can lead to apoptosis. PM can activate the Toll-like receptor (TLR), leading to activation of the mitogen-activated protein kinase (MAPK) pathway and the nuclear factor kappa B (NF-κB) pathway. This, in turn, leads to an upregulation of gene expression of cytokines such as tumor necrosis factor (*TNF*), interleukin 1 alpha (*IL1A*), interleukin 1 beta (*IL1B*), as well as, chemokines such as C-X-C motif chemokine ligand 8 (*CXCL8*), adhesion molecules such as intercellular adhesion molecule 1 (*ICAM1*), enzymes such as cyclooxygenase 2 (*COX2*) and inducible nitric oxide synthase (*iNOS*), and matrix metalloproteases (*MMP*s). Fig. A is adapted with permission from van Smeden et al. and elements have been used for Fig. B [247]

It was shown in vitro and in vivo that PM-induced skin barrier dysfunction, oxidative stress, and inflammation are interconnected and exaggerate each other (see the working model in Fig. 2).

### Protective solutions against PM-induced skin damage

Understanding the underlying mechanisms of PM’s adverse effects on human skin is crucial for the development of novel protective dermato-cosmetic technologies. To date, whereas only few technologies were developed to specifically target cutaneous damages induced by air pollutants in general, protection benefits against PM-induced skin damages were found for well-known cosmetic ingredients. Most of them are antioxidant based and aim to counteract the oxidative stress induced by PM and prevent the activation of the linked pro-inflammatory response. However, in most cases, the exact mechanism of action of these ingredients is yet to be elucidated.

Several active compounds were found to protect skin against PM-induced damage by inhibiting the expression of AhR. For example, N-acetylcysteine (NAC), was shown to suppress the expression of AhR and its nuclear translocation in human keratinocytes and attenuated the increase of intracellular ROS levels upon PM-treatment [124]. In addition, topical NAC application in mice, showed to protect against ROS-AhR dependent PM-damage in the skin, by protecting from a PM-induced hyperkeratotic epidermis [124]. In another study, the inhibition of the AhR with benzylidene dimethoxydimethylindanone in PM-treated keratinocytes and fibroblasts, resulted in decreased transcription of one of its target genes *CYP1A1* and in a decreased subsequent pro-inflammatory response by a decrease in MMP-1 protein expression [172]. *Camellia japonica* flower extract was shown to have antioxidant properties in human fibroblasts by decreasing PM-induced intracellular ROS levels and subsequently inhibiting AhR activity and CYP1A1 transcription [119]. In addition, this extract has shown to protect from PM-induced lipid peroxidation in an ex vivo human skin model [119].

The potent antioxidant vitamin E was shown to rescue PM-induced damage in keratinocytes by partially restoring the expression of proteins that are important for proliferation, differentiation, and desmosomal integrity, among others [127]. Resveratrol, another strong antioxidant, was shown to repress the PM-induced pro-inflammatory response in primary keratinocytes [257].

Alginate extracted from *Sargassum horneri* was shown to have metal ion chelating properties by decreasing the concentration of PM metal traces (i.e., Pb, Ca, Sr, Ba, and Mg) in keratinocytes in a dose-dependent manner [231]. This led to a decrease in intracellular ROS levels and downstream inactivation of the NF-κB and MAPK signaling pathways [231].

Ethanol extract of *Cornus officinalis* fruit (EECF) [183], a ROS scavenger, showed an antioxidant potential in HaCaT cells, by protecting against PM-induced DNA damage, lipid peroxidation, and protein carbonylation. It also prevented increases in cellular calcium levels and inhibited apoptosis [183]. A pre-treatment with *Astragali Radix* or its main compound formononetin resulted in the protection against PM-induced decrease in keratinocyte proliferation and differentiation markers and blocked PM-induced apoptosis in a 2D and 3D skin model [125]. Green tea extract has shown antioxidant and anti-inflammatory properties in keratinocytes [134]. Additionally, it was shown to restore the PM-induced unbalance in cholesterol metabolism in a 3D HSE model [134]. Moreover, the major polyphenolic antioxidant compound of green tea, epigallocatechin-3-gallate (EGCG), has shown ROS scavenging properties by decreasing PM-induced ROS levels in fibroblasts [136]. In turn, this resulted in restoring elastase and collagenase activity and the expression level of several MMPs. Treatment with EGCG blocked the nuclear translocation of p65 as well as the phosphorylations of several MAPKs in a dose-dependent manner [136]. A slight decrease of PM-induced ROS by EGCG in keratinocytes was shown to result in the balance restoration of TNF-α, IL-1β, IL-6, IL-8, and MMP-1 mRNA and protein levels [173].

2′-Fucosyllactose (2FL) was tested in HaCaT and shown to have protective effects against PM_10_-induced pro-inflammatory response [258]. Eckol, a phlorotannin extracted from seaweed, has shown antioxidant properties in keratinocytes [182]. Treatment with eckol reduced PM-induced intracellular ROS levels, leading to maintenance of the mitochondrial function and protecting from MAPK activation and PM-induced apoptosis [182]. Additionally, other algae extracts have shown to protect the skin from damage in vitro, such as *Ecklonia cava* extract and its compounds dieckol [126], fucosterol [137], diphlorethohydroxycarmalol [168], afzelin [180], and eupafolin [179]. Ethyl 2,4-dicarboethoxy pantothenate, which is a derivative of pantothenic acid, was shown to reduce to PM-induced cellular damage [171].

Niacinamide, a compound that is often used in various cosmetics, was shown to protect keratinocytes from PM-induced oxidative stress [184]. Other reducing agents found to protect the skin against PM are 3,4-dicaffeoylquinic acid [186], 7,3′,4′-trihydroxyisoflavone cyclodextrin inclusion complex [142], isovitexin [176], ginsenoside Rb1 [175], chitosan [259], and SIG-1273, a isoprenylcysteine small molecule [228].

Nanoparticle technologies, such as 734THI nanoparticle powder [230], eupafolin nanoparticles [181], and nanomaterial fullerene derivate fullerenol [143], were also considered as new players in the field of airborne PM protection.

Although most of the studies were carried out using an active ingredient only, protective effects against PM were demonstrated also from finished formulations. For example, a novel water-in-oil emulsion with a lecithin-modified bentonite, applied topically on a HEE 3D model, prevented skin damage from urban dust and cedar pollen, as indicated by the decrease in MMP-1 and IL-8 protein secretion [129].

## Future directions

2D skin models lack the barrier properties that 3D skin models have, and PM treatment can result in an overestimation of the real effect. Since the greater part of PM research on skin has been done using 2D models, it is interesting though challenging to translate the results into 3D models and even in vivo outcomes. In a recent study, the irritation potency of several compounds, has been compared in both 2D and 3D models [260]. By screening 451 compounds in 2D models, they were able to select 46 compounds that showed cytotoxicity. From the 46 compounds, only seven compounds showed a cytotoxic potential in a HEE model and only one compound in a HSE model. This result underlines that 2D usually gives an overestimation of the assessed effect compared to 3D skin models. However, it can be used as a tool to gain preliminary knowledge on an expected response towards cytotoxicity, for example. The use of 3D skin models, such as HEE and HSE models, as an alternative to animal testing is recommended when testing the cutaneous effects of PM on skin. However, one must keep in mind that 3D skin models possess weaker barrier properties compared to the native skin. More advanced and relevant 3D skin models are developed that include immune cells, melanocytes, sebocytes, and endothelial cells, stimuli for lipid synthesis [261], and cultivation conditions closer to the real-life situation [262]; hence a complete immune system [263], vascularization [264], and barrier properties similar to human skin are the main elements that current models are lacking [265]. Skin surface lipids (e.g., sebum) were not considered to date in 3D skin models utilized for PM-testing. As aforementioned, the skin surface lipids are prone to be oxidized by PAH-species adsorbed on PM, and their oxidation byproducts were shown to affect the skin. Therefore, it could be of high relevance to consider artificial sebum with a relevant lipid composition in 3D skin models to further understand the detrimental effects of PM on the skin [266, 267]. Moreover, testing of diseased skin models, such as AE 3D skin models, could offer more understanding of the underlying mechanisms of symptom aggravation upon exposure to PM [265, 268, 269]. For the assessment of PM effects on skin aging, advanced skin models for skin aging research can be employed [270].

When using 3D skin models, it is recommended to apply PM topically, that is a closer to real-life condition compared to systemic treatment. Additionally, the chemical interactions of PM with the skin and the PM properties upon interaction with the skin should be further investigated to perform exposure experiments that have a higher physiological relevance. It is important to study the correlation between PM source and composition on the effects that it has on the skin.

Most of the PM is coming from reference standards that can be either outdated (i.e., collected in 1976) or coming from a certain geographical region, leading to a wrong generalization of the current state of airborne PM. An example is testing of diesel particles: due to the continuously improving filtering systems of diesel cars, the emitted particles for the modern filter-equipped diesel cars are producing far less PM compared to gasoline cars [271]. Although airborne PM is decreasing in the developed world, due to cars being equipped with improved filtering systems, the air quality in developing countries is a lot worse [272], due to among other things, the lack of sufficient funds for these new filtering technologies. PM collected from different cities in a broader selection of the world, would give a better understanding of the real effects of PM on the skin.

PM is only one of the major hazardous elements of air pollution, and other elements, such as ozone and UV radiation, are important to investigate as well. Combined exposures of skin models, including several pollutants, would allow for a more relevant exposure scenario and give more insight into the interactions between the pollutants and the skin; hence synergistic damage of these pollutants is suggested [273, 274].

Taken together, these future directions could contribute to create recommendations for regulations to reduce airborne PM and for the dermato-cosmetic industry to design novel solutions for improved skin protection against air pollution.

## Conclusions

The effects of airborne PM on skin have been intensively studied, but the majority is done in 2D skin models, especially in HaCaT cells and primary keratinocytes. Those cells are exposed to dispersed PM in cell culture media and thus reflect the real-life situation to a lesser extent, where the outer skin layer is consisting of the SC and exposed to air.

The results summarized do suggest not to limit our investigations to the assessment of oxidative stress and inflammation through ROS production and pro-inflammatory protein expression and secretion, respectively, but also to include implications in the complete mitochondrial respiratory chain, skin integrity, skin barrier function, epidermal differentiation and proliferation to the assessment.

Airborne PM has shown to affect several distinct cellular processes to a high degree, making it a very intriguing subject to study. Investigating these effects on a model that better resembles the complexity of human skin, a HSE or HEE model, or more advanced 3D skin models, will further reveal the involved cellular processes and effects on the health of human skin. By targeting these processes in both healthy and diseased skin, skin damage could be prevented and possibly cured by providing new solutions for the development of skincare products.

## Data Availability

Not applicable.

## Abbreviations

AE: Atopic eczema
AhR: Aryl hydrocarbon receptor
AhRR: AhR repressor
AMP: Anti-microbial peptide
ARE: Antioxidant response element
ARNT: AhR nuclear translocator
AP-1: Adaptor protein 1
BaP: Benzo[a]pyrene
BrdU: 5-bromo-2′-deoxyuridine
CAP: Concentrated ambient air particles
CHOP: CCAAT-enhancer-binding protein homologous protein
CRM: Certified reference material
COX2: Cyclooxygenase 2
CYP1A1: Cytochrome P450 family 1 subfamily A member 1
DC: Dendritic cell
DEP: Diesel exhaust particles
DMBA: Dimethylbenz [a]anthracene
EGFR: Epidermal growth factor receptor
ER: Endoplasmic reticulum
ERK: Extracellular-signal-regulated kinase
ERM: European reference material
GRP78: ER chaperone BiP
GST: Glutathione S-transferase
HEE: Human epidermal equivalent
HMOX1: Heme oxygenase 1
HNE: 4-hydroxy-2-nonenal
HSE: Human skin equivalent
IgE: Immunoglobulin E
IL: Interleukin
IRE1α: Inositol-requiring 1 alpha
IRF: Interferon regulatory factor
JNK: c-Jun-NH2-terminal kinase
JRC: Joint research centre
LB: Lamellar body
LC: Langerhans cell
LC3B-II: Light-chain 3B II
LPS: Lipopolysaccharide
MDA: Malondialdehyde
MAPK: Mitogen-activated protein kinase
MMP: Matrix metalloproteinase
MyD88: Myeloid differentiation primary response 88
NADPH: Nicotinamide adenine dinucleotide phosphate
Nrf2: Nuclear factor erythroid 2-related factor 2
NF-κB: Nuclear factor kappa B
NHDF: Normal human dermal fibroblasts
NHEK: Normal human epidermal keratinocytes
NLPR3: NOD-, LRR- and pyrin domain-containing protein 3
NLR: Leucine-rich repeat-containing
NOX: NADPH oxidase
NQO1: NAD(P) H quinone dehydrogenase 1
PAH: Polycyclic hydrocarbons
PAMP: Pathogen-associated molecular patterns
PARP: Poly (ADP-ribose) polymerase
PERK: Protein kinase-like ER kinase
PM: Particulate matter
PRR: Pattern recognition receptor
ROS: Reactive oxygen species
SB: Stratum basale
SC: Stratum corneum
SG: Stratum granulosum
SS: Stratum spinosum
TCDD: 2,3,7,8-tetrachlorodibenzodioxin
TEWL: Trans-epidermal water loss
TLR: Toll-like receptor
TNF-α: Tumor necrosis factor α
TRIF: TIR-domain-containing adapter-inducing interferon-β
XRE: Xenobiotic response element

## References

[1] World Health Organization. Ambient air pollution: A global assessment of exposure and burden of disease. WHO [Internet]. 2016 [cited 2019 Jul 16]; Available from: https://www.who.int/phe/publications/air-pollution-global-assessment/en/.

[2] Health Effects Institute. State of Global Air 2019 [Internet]. 2019 [cited 2020 Jan 31]. Available from: https://www.stateofglobalair.org/report.

[3] Kampa M, Castanas E. Human health effects of air pollution. Environ Pollut. 2008;151:362–7. 10.1016/j.envpol.2007.06.012 17646040

[4] Karagulian F, Belis CA, Dora CFC, Prüss-Ustün AM, Bonjour S, Adair-Rohani H, et al. Contributions to cities’ ambient particulate matter (PM): A systematic review of local source contributions at global level. Atmos Environ. 2015;120:475–83.

[5] Adams K, Greenbaum DS, Shaikh R, van Erp AM, Russell AG. Particulate matter components, sources, and health: Systematic approaches to testing effects. J Air Waste Manage Assoc. 2015;65:544–58. 10.1080/10962247.2014.1001884 25947313

[6] US Environmental Protection Agency (EPA). Particulate Matter (PM) Pollution [Internet]. 2020 [cited 2020 Jan 31]. Available from: https://www.epa.gov/pm-pollution.

[7] de Kok TMCM, Driece HAL, Hogervorst JGF, Briedé JJ. Toxicological assessment of ambient and traffic-related particulate matter: A review of recent studies. Mutat Res - Rev Mutat Res. 2006;613:103–22. 10.1016/j.mrrev.2006.07.001 16949858

[8] Phalen RF. The particulate air pollution controversy. Part. Air Pollut. Controv. 2002.

[9] Oberdörster G. Pulmonary effects of inhaled ultrafine particles. Int Arch Occup Environ Health. 2000;74:1–8. 10.1007/s004200000185 11196075

[10] Tobiszewski M, Namieśnik J. PAH diagnostic ratios for the identification of pollution emission sources. Environ Pollut. 2012;162:110–9. 10.1016/j.envpol.2011.10.025 22243855

[11] Idowu O, Semple KT, Ramadass K, O’Connor W, Hansbro P, Thavamani P. Beyond the obvious: Environmental health implications of polar polycyclic aromatic hydrocarbons. Environ Int. 2019;123:543–57. 10.1016/j.envint.2018.12.051 30622079

[12] Holme JA, Brinchmann BC, Refsnes M, Låg M, Øvrevik J. Potential role of polycyclic aromatic hydrocarbons as mediators of cardiovascular effects from combustion particles. Environ Heal A Glob Access Sci Source. 2019;18:74. 10.1186/s12940-019-0514-2 PMC6704565 31439044

[13] Boström CE, Gerde P, Hanberg A, Jernström B, Johansson C, Kyrklund T, et al. Cancer risk assessment, indicators, and guidelines for polycyclic aromatic hydrocarbons in the ambient air. Environ Health Perspect. 2002;110:451–88. 10.1289/ehp.110-1241197 PMC1241197 12060843

[14] Sydbom A, Blomberg A, Parnia S, Stenfors N, Sandström T, Dahlén SE. Health effects of diesel exhaust emissions. Eur Respir J. 2001;17:733–46. 10.1183/09031936.01.17407330 11401072

[15] Newby DE, Mannucci PM, Tell GS, Baccarelli AA, Brook RD, Donaldson K, et al. Expert position paper on air pollution and cardiovascular disease. Eur Heart J. 2015;36:83–93. 10.1093/eurheartj/ehu458 PMC6279152 25492627

[16] Holgate ST. ‘Every breath we take: the lifelong impact of air pollution’ – a call for action. Clin Med (Northfield Il). 2017;17:8–12. 10.7861/clinmedicine.17-1-8 PMC6297602 28148571

[17] Schraufnagel DE, Balmes JR, Cowl CT, De Matteis S, Jung SH, Mortimer K, et al. Air pollution and noncommunicable diseases: A review by the Forum of International Respiratory Societies’ Environmental Committee, Part 2: Air pollution and organ systems. Chest. 2019;155:417–26. 10.1016/j.chest.2018.10.041 PMC6904854 30419237

[18] Mancebo SE, Wang SQ. Recognizing the impact of ambient air pollution on skin health. J Eur Acad Dermatology Venereol. 2015;29:2326–32. 10.1111/jdv.13250 PMC5916788 26289769

[19] Krutmann J, Bouloc A, Sore G, Bernard BA, Passeron T. The skin aging exposome. J Dermatol Sci. 2017;85:152–61. 10.1016/j.jdermsci.2016.09.015 27720464

[20] Krutmann J, Liu W, Li L, Pan X, Crawford M, Sore G, et al. Pollution and skin: From epidemiological and mechanistic studies to clinical implications. J Dermatol Sci. 2014;76:163–8. 10.1016/j.jdermsci.2014.08.008 25278222

[21] Araviiskaia E, Berardesca E, Bieber T, Gontijo G, Sanchez Viera M, Marrot L, et al. The impact of airborne pollution on skin. J Eur Acad Dermatology Venereol. 2019;33:1496–505. 10.1111/jdv.15583 PMC6766865 30897234

[22] van Smeden J. A breached barrier: Analysis of stratum corneum lipids and their role in eczematous patients [Internet]. Leiden Academic Center for Drug Research (LACDR), Faculty of Science, Leiden University; 2013 [cited 2020 Feb 5]. Available from: https://openaccess.leidenuniv.nl/handle/1887/20998.

[23] Keene DR, Marinkovich MP, Sakai LY. Immunodissection of the connective tissue matrix in human skin. Microsc Res Tech. 1997;38:394–406. 10.1002/(SICI)1097-0029(19970815)38:4<394::AID-JEMT7>3.0.CO;2-J 9297689

[24] Moore KL, Dalley AF, Agur AM. Clinically orientated anatomy. 2010.

[25] Kielty CM, Shuttleworth CA. Microfibrillar elements of the dermal matrix. Microsc Res Tech. 1997;38:413–27. 10.1002/(SICI)1097-0029(19970815)38:4<413::AID-JEMT9>3.0.CO;2-J 9297691

[26] Frantz C, Stewart KM, Weaver VM. The extracellular matrix at a glance. J Cell Sci. 2010;123:4195–200. 10.1242/jcs.023820 PMC2995612 21123617

[27] Williams IR, Kupper TS. Immunity at the surface: Homeostatic mechanisms of the skin immune system. Life Sci. 1996;58:1485–507. 10.1016/0024-3205(96)00042-2 8649179

[28] Nestle FO, Di Meglio P, Qin JZ, Nickoloff BJ. Skin immune sentinels in health and disease. Nat Rev Immunol. 2009;9:679–91. 10.1038/nri2622 PMC2947825 19763149

[29] McLafferty E, Hendry C, Farley A. The integumentary system: anatomy, physiology and function of skin. Nurs Stand. 2012;27:35–42. 10.7748/ns2012.10.27.7.35.c9358 23248884

[30] Elias MP, Feingold KR, Fartasch M. The epidermal lamellar body as a multifunctional secretory organelle. Ski Barrier. 2006. p. 261–72.

[31] Feingold KR. Lamellar bodies: The key to cutaneous barrier function. J Invest Dermatol. 2012;132:1951–3. 10.1038/jid.2012.177 22797297

[32] Arda O, Göksügür N, Tüzün Y. Basic histological structure and functions of facial skin. Clin Dermatol. Elsevier; 2014;32:3–13. 10.1016/j.clindermatol.2013.05.021 24314373

[33] Bouwstra JA, Honeywell-Nguyen PL, Gooris GS, Ponec M. Structure of the skin barrier and its modulation by vesicular formulations. Prog Lipid Res. 2003;42:1–36. 10.1016/s0163-7827(02)00028-0 12467638

[34] Blair C. Morphology and thickness of the human stratum corneum. Br J Dermatol. 1968;80:430–6. 10.1111/j.1365-2133.1968.tb11978.x 4233061

[35] Holbrook KA, Odland GF. Regional differences in the thickness (cell layers) of the human stratum corneum: an ultrastructural analysis. J Invest Dermatol. 1974;62:415–22. 10.1111/1523-1747.ep12701670 4820685

[36] Rousselle P, Gentilhomme E, Neveux Y. Markers of epidermal proliferation and differentiation. Agache’s Meas Ski Non-invasive Investig Physiol Norm Constants Second Ed. 2017. p. 407–15.

[37] Kim KE, Cho D, Park HJ. Air pollution and skin diseases: Adverse effects of airborne particulate matter on various skin diseases. Life Sci. 2016;152:126–34. 10.1016/j.lfs.2016.03.039 27018067

[38] Valacchi G, Sticozzi C, Pecorelli A, Cervellati F, Cervellati C, Maioli E. Cutaneous responses to environmental stressors. Ann N Y Acad Sci. 2012;1271:75–81. 10.1111/j.1749-6632.2012.06724.x PMC3495295 23050967

[39] McDaniel D, Farris P, Valacchi G. Atmospheric skin aging—Contributors and inhibitors. J Cosmet Dermatol. 2018;17:124–37. 10.1111/jocd.12518 29575554

[40] Vierkötter A, Schikowski T, Ranft U, Sugiri D, Matsui M, Krämer U, et al. Airborne particle exposure and extrinsic skin aging. J Invest Dermatol. 2010;130:2719–26. 10.1038/jid.2010.204 20664556

[41] Schikowski T, Krutmann J. Luftverschmutzung (Feinstaub, Stickstoffdioxid) und Hautalterung. Der Hautarzt. 2019;70:158–62. 10.1007/s00105-018-4338-8 30627745

[42] Peng F, Xue C-H, Hwang SK, Li W-H, Chen Z, Zhang J-Z. Exposure to fine particulate matter associated with senile lentigo in Chinese women: A cross-sectional study. J Eur Acad Dermatology Venereol. 2017;31:355–60. 10.1111/jdv.13834 PMC6084310 27593207

[43] Yaar M, Eller MS, Gilchrest BA. Fifty years of skin aging. J Investig Dermatology Symp Proc. 2002. p. 51–8. 10.1046/j.1523-1747.2002.19636.x 12518793

[44] Vogeley C, Esser C, Tüting T, Krutmann J, Haarmann-Stemmann T. Role of the aryl hydrocarbon receptor in environmentally induced skin aging and skin Carcinogenesis. Int J Mol Sci. 2019;20:6005. 10.3390/ijms20236005 PMC6928879 31795255

[45] Parrado C, Mercado-Saenz S, Perez-Davo A, Gilaberte Y, Gonzalez S, Juarranz A. Environmental stressors on skin aging. Mechanistic insights. Front Pharmacol. 2019;10:759. 10.3389/fphar.2019.00759 PMC6629960 31354480

[46] Burke KE. Mechanisms of aging and development—A new understanding of environmental damage to the skin and prevention with topical antioxidants. Mech Ageing Dev. 2018;172:123–30. 10.1016/j.mad.2017.12.003 29287765

[47] Radespiel-Tröger M, Geiss K, Twardella D, Maier W, Meyer M. Cancer incidence in urban, rural, and densely populated districts close to core cities in Bavaria, Germany. Int Arch Occup Environ Health. 2018;91:155–74. 10.1007/s00420-017-1266-3 29027001

[48] Datzmann T, Markevych I, Trautmann F, Heinrich J, Schmitt J, Tesch F. Outdoor air pollution, green space, and cancer incidence in saxony: A semi-individual cohort study. BMC Public Health. 2018;18:715. 10.1186/s12889-018-5615-2 PMC5994126 29884153

[49] Weidinger S, Novak N. Atopic Dermatitis. Lancet. 2016;387:1109–22. 10.1016/S0140-6736(15)00149-X 26377142

[50] Brunello L. Atopic Dermatitis. Nat Rev Dis Prim. 2018;4:2. 10.1038/s41572-018-0004-9 29930279

[51] Tsakok T, Woolf R, Smith CH, Weidinger S, Flohr C. Atopic dermatitis: the skin barrier and beyond. Br J Dermatol. 2019;180:464–74. 10.1111/bjd.16934 29969827

[52] Ahn K. The role of air pollutants in atopic dermatitis. J Allergy Clin Immunol. 2014;134:993–9. 10.1016/j.jaci.2014.09.023 25439225

[53] Ngoc L, Park D, Lee Y, Lee Y-C. Systematic review and meta-analysis of human skin diseases due to particulate matter. Int J Environ Res Public Health. 2017;14:1458. 10.3390/ijerph14121458 PMC5750877 29186837

[54] Lee J-Y, Lamichhane D, Lee M, Ye S, Kwon J-H, Park M-S, et al. Preventive effect of residential green space on infantile atopic dermatitis associated with prenatal air pollution exposure. Int J Environ Res Public Health. 2018;15:102. 10.3390/ijerph15010102 PMC5800201 29315266

[55] Hüls A, Klümper C, MacIntyre EA, Brauer M, Melén E, Bauer M, et al. Atopic dermatitis: Interaction between genetic variants of GSTP1 , TNF , TLR2 , and TLR4 and air pollution in early life. Pediatr Allergy Immunol. 2018;29:596–605. 10.1111/pai.12903 29624745

[56] Kim Y-M, Kim J, Jung K, Eo S, Ahn K. The effects of particulate matter on atopic dermatitis symptoms are influenced by weather type: Application of spatial synoptic classification (SSC). Int J Hyg Environ Health. 2018;221:823–9. 10.1016/j.ijheh.2018.05.006 29853291

[57] Schnass W, Hüls A, Vierkötter A, Krämer U, Krutmann J, Schikowski T. Traffic-related air pollution and eczema in the elderly: Findings from the SALIA cohort. Int J Hyg Environ Health. 2018;221:861–7. 10.1016/j.ijheh.2018.06.002 29908909

[58] Noh SR, Kim J-S, Kim E-H, Jeon B-H, Kim J-H, Kim Y-M, et al. Spectrum of susceptibility to air quality and weather in individual children with atopic dermatitis. Pediatr Allergy Immunol. 2019;30:179–87. 10.1111/pai.13005 30428138

[59] Belugina IN, Yagovdik NZ, Belugina OS, Belugin SN. Outdoor environment, ozone, radionuclide-associated aerosols and incidences of infantile eczema in minsk, belarus. J Eur Acad Dermatology Venereol. 2018;32:1977–85. 10.1111/jdv.15063 29730889

[60] Guo Q, Xiong X, Liang F, Tian L, Liu W, Wang Z, et al. The interactive effects between air pollution and meteorological factors on the hospital outpatient visits for atopic dermatitis in Beijing, China: A time-series analysis. J Eur Acad Dermatology Venereol. 2019;33:2362–70. 10.1111/jdv.15820 31325384

[61] Krämer U, Behrendt H. Luftverschmutzung und atopisches Ekzem. Der Hautarzt. 2019;70:169–84. 10.1007/s00105-018-4330-3 30659336

[62] Yang HJ, Lee SY, Suh DI, Shin YH, Kim BJ, Seo JH, et al. The Cohort for Childhood Origin of Asthma and allergic diseases (COCOA) study: Design, rationale and methods. BMC Pulm Med. 2014;14:109. 10.1186/1471-2466-14-109 PMC4099383 24990471

[63] Kelleher M, Dunn-Galvin A, Hourihane JOB, Murray D, Campbell LE, McLean WHI, et al. Skin barrier dysfunction measured by transepidermal water loss at 2 days and 2 months predates and predicts atopic dermatitis at 1 year. J Allergy Clin Immunol. 2015;135:930–935.e1. 10.1016/j.jaci.2014.12.013 PMC4382348 25618747

[64] Lee E, Lee S-Y, Kim H-C, Choi KY, Kim H-B, Park MJ, et al. Prenatal particulate matter exposure with skin barrier dysfunction affects offspring’s atopic dermatitis: COCOA study. J Allergy Clin Immunol Pract. 2020;8. 10.1016/j.jaip.2020.01.040 32006726

[65] Wei T, Tang M. Biological effects of airborne fine particulate matter (PM2.5) exposure on pulmonary immune system. Environ Toxicol Pharmacol. 2018;60:195–201. 10.1016/j.etap.2018.04.004 29734103

[66] Hassoun Y, James C, Bernstein DI. The effects of air pollution on the development of atopic disease. Clin Rev Allergy Immunol. 2019;57:403–14. 10.1007/s12016-019-08730-3 PMC8215519 30806950

[67] Glencross DA, Ho TR, Camiña N, Hawrylowicz CM, Pfeffer PE. Air pollution and its effects on the immune system. Free Radic Biol Med. 2020;151:56–68. 10.1016/j.freeradbiomed.2020.01.179 32007522

[68] Dong Y mao, Liao L ying, Li L, Yi F, Meng H, He Y fan, et al. Skin inflammation induced by ambient particulate matter in China. Sci Total Environ. 2019;682:364–73. 10.1016/j.scitotenv.2019.05.155 31125750

[69] Hendricks AJ, Eichenfield LF, Shi VY. The impact of airborne pollution on atopic dermatitis—A literature review. Br J Dermatol. 2019;18781. 10.1111/bjd.18781 31794065

[70] Kantor R, Silverberg JI. Environmental risk factors and their role in the management of atopic dermatitis. Expert Rev Clin Immunol. 2017;13:15–26. 10.1080/1744666X.2016.1212660 PMC5216178 27417220

[71] Niwa Y, Sumi H, Kawahira K, Terashima T, Nakamura T, Akamatsu H. Protein oxidative damage in the stratum corneum: Evidence for a link between environmental oxidants and the changing prevalence and nature of atopic dermatitis in Japan. Br J Dermatol. 2003;149:248–54. 10.1046/j.1365-2133.2003.05417.x 12932228

[72] Kabashima K, Otsuka A, Nomura T. Linking air pollution to atopic dermatitis. Nat Immunol. 2017;18:5–6. 10.1038/ni.3615 27984566

[73] Hidaka T, Ogawa E, Kobayashi EH, Suzuki T, Funayama R, Nagashima T, et al. The aryl hydrocarbon receptor AhR links atopic dermatitis and air pollution via induction of the neurotrophic factor artemin. Nat Immunol. 2017;18:64–73. 10.1038/ni.3614 27869817

[74] Van Smeden J, Janssens M, Boiten WA, Van Drongelen V, Furio L, Vreeken RJ, et al. Intercellular skin barrier lipid composition and organization in netherton syndrome patients. J Invest Dermatol. 2014;134:1238–45. 10.1038/jid.2013.517 24292773

[75] Wolf R, Orion E, Ruocco E, Ruocco V. Abnormal epidermal barrier in the pathogenesis of psoriasis. Clin Dermatol. 2012;30:323–8. 10.1016/j.clindermatol.2011.08.022 22507047

[76] Standard Reference Materials | NIST [Internet]. [cited 2019 Jul 19]. Available from: https://www.nist.gov/srm.

[77] Certified Reference Materials catalogue of the JRC [Internet]. [cited 2019 Jul 19]. Available from: https://crm.jrc.ec.europa.eu/.

[78] Mori I, Sun Z, Ukachi M, Nagano K, McLeod CW, Cox AG, et al. Development and certification of the new NIES CRM 28: Urban aerosols for the determination of multielements. Anal Bioanal Chem. 2008;391:1997–2003. 10.1007/s00216-008-2076-y 18414834

[79] Abd E, Yousef SA, Pastore MN, Telaprolu K, Mohammed YH, Namjoshi S, et al. Skin models for the testing of transdermal drugs. Clin Pharmacol. 2016;8:163–76. 10.2147/CPAA.S64788 PMC5076797 27799831

[80] Todo H. Transdermal permeation of drugs in various animal species. Pharmaceutics. 2017;9:33. 10.3390/pharmaceutics9030033 PMC5620574 28878145

[81] Montagna W, And PD, Yen JS. The skin of domestic pig. J Invest Dermatol. 1964;42:11–21. 14209446

[82] Sullivan TP, Eaglstein WH, Davis SC, Mertz P. The pig as a model for human wound healing. Wound Repair Regen. 2001;9:66–76. 10.1046/j.1524-475x.2001.00066.x 11350644

[83] Debeer S, Le Luduec JB, Kaiserlian D, Laurent P, Nicolas JF, Dubois B, et al. Comparative histology and immunohistochemistry of porcine versus human skin. Eur J Dermatology. 2013;23:456–66. 10.1684/ejd.2013.2060 24047577

[84] Kong R, Bhargava R. Characterization of porcine skin as a model for human skin studies using infrared spectroscopic imaging. Analyst. 2011;136:2359–66. 10.1039/c1an15111h 21509377

[85] Summerfield A, Meurens F, Ricklin ME. The immunology of the porcine skin and its value as a model for human skin. Mol Immunol. 2015;66:14–21. 10.1016/j.molimm.2014.10.023 25466611

[86] Williams MG, Hunter R. Studies on epidermal regeneration by means of the strip method. J Invest Dermatol. 1957;29:407–13. 10.1038/jid.1957.116 13502596

[87] Gerritsen MJP, van Erp PEJ, van Vlijmen-Willems IMJJ, Lenders LTM, van de Kerkhof PCM. Repeated tape stripping of normal skin: a histological assessment and comparison with events seen in psoriasis. Arch Dermatol Res. 1994;286:455–61. 10.1007/BF00371571 7532389

[88] Ghadially R, Brown BE, Sequeira-Martin SM, Feingold KR, Elias PM. The aged epidermal permeability barrier. Structural, functional, and lipid biochemical abnormalities in humans and a senescent murine model. J Clin Invest. 1995;95:2281–90. 10.1172/JCI117919 PMC295841 7738193

[89] Dickel H, Goulioumis A, Gambichler T, Fluhr JW, Kamphowe J, Altmeyer P, et al. Standardized tape stripping: a practical and reproducible protocol to uniformly reduce the stratum corneum. Ski Pharmacol Physiol. 2010;23:259–65. 10.1159/000314700 20484967

[90] Gao Y, Wang X, Chen S, Li S, Liu X. Acute skin barrier disruption with repeated tape stripping: An in vivo model for damage skin barrier. Ski Res Technol. 2013;19:162–8. 10.1111/srt.12028 23279155

[91] Boiten WA, Berkers T, Absalah S, Van Smeden J, Lavrijsen APM, Bouwstra JA. Applying a vernix caseosa based formulation accelerates skin barrier repair by modulating lipid biosynthesis. J Lipid Res. 2018;59:250–60. 10.1194/jlr.M079186 PMC5794420 29217624

[92] Berkers T, Boiten WA, Absalah S, van Smeden J, Lavrijsen APM, Bouwstra JA. Compromising human skin in vivo and ex vivo to study skin barrier repair. Biochim Biophys Acta - Mol Cell Biol Lipids. 2019;1864:1103–8. 10.1016/j.bbalip.2019.04.005 31002944

[93] Matsuda H, Watanabe N, Geba GP, Sperl J, Tsudzuki M, Hiroi J, et al. Development of atopic dermatitis-like skin lesion with IgE hyperproduction in NC/Nga mice. Int Immunol. 1997;9:461–6. 10.1093/intimm/9.3.461 9088984

[94] Matsumoto M, Ra C, Kawamoto K, Sato H, Itakura A, Sawada J, et al. IgE hyperproduction through enhanced tyrosine phosphorylation of Janus kinase 3 in NC/Nga mice, a model for human atopic dermatitis. J Immunol. 1999;162:1056–63. 9916733

[95] Suto H, Matsuda H, Mitsuishi K, Hira K, Uchida T, Unno T, et al. NC/Nga mice: A mouse model for atopic dermatitis. Int Arch Allergy Immunol. 1999;120:70–5. 10.1159/000053599 10529609

[96] Vestergaard C, Yoneyama H, Murai M, Nakamura K, Tamaki K, Terashima Y, et al. Overproduction of Th2-specific chemokines in NC/Nga mice exhibiting atopic dermatitis-like lesions. J Clin Invest. 1999;104:1097–105. 10.1172/JCI7613 PMC408579 10525048

[97] Rollin BE. Toxicology and new social ethics for animals. Toxicol Pathol. 2003;31:128–31. 10.1080/01926230390175011 12597441

[98] Ranganatha N, Kuppast IJ. A review on alternatives to animal testing methods in drug development. Int J Pharm Pharm Sci. 2012;4:28–32.

[99] Doke SK, Dhawale SC. Alternatives to animal testing: A review. Saudi Pharm J. 2015;23:223–9. 10.1016/j.jsps.2013.11.002 PMC4475840 26106269

[100] Boukamp P, Petrussevska RT, Breitkreutz D, Hornung J, Markham A, Fusenig NE. Normal keratinization in a spontaneously immortalized aneuploid human keratinocyte cell line. J Cell Biol. 1988;106:761–71. 10.1083/jcb.106.3.761 PMC2115116 2450098

[101] Soboleva AG, Zolotarenko AD, Sobolev V V., Bruskin SA, Piruzian ES, Mezentsev A V. Genetically predetermined limitation in HaCaT cells that affects their ability to serve as an experimental model of psoriasis. Russ J Genet. 2014;50:1081–9. 25720254

[102] Seo MD, Kang TJ, Lee CH, Lee AY, Noh M. HaCa T keratinocytes and primary epidermal keratinocytes have different transcriptional profiles of cornified envelope-associated genes to T helper cell cytokines. Biomol Ther. 2012;20:171–6. 10.4062/biomolther.2012.20.2.171 PMC3792214 24116291

[103] Sprenger A, Weber S, Zarai M, Engelke R, Nascimento JM, Gretzmeier C, et al. Consistency of the proteome in primary human keratinocytes with respect to gender, age, and skin localization. Mol Cell Proteomics. 2013;12:2509–21. 10.1074/mcp.M112.025478 PMC3769327 23722187

[104] Sun T, Jackson S, Haycock JW, MacNeil S. Culture of skin cells in 3D rather than 2D improves their ability to survive exposure to cytotoxic agents. J Biotechnol. 2006;122:372–81. 10.1016/j.jbiotec.2005.12.021 16446003

[105] Chen L, Wu M, Jiang S, Zhang Y, Li R, Lu Y, et al. Skin toxicity assessment of silver nanoparticles in a 3D epidermal model compared to 2D keratinocytes. Int J Nanomedicine. 2019;14:9707–19. 10.2147/IJN.S225451 PMC6910103 31849463

[106] Niehues H, Bouwstra JA, El Ghalbzouri A, Brandner JM, Zeeuwen PLJM, van den Bogaard EH. 3D skin models for 3R research: The potential of 3D reconstructed skin models to study skin barrier function. Exp Dermatol. 2018;27:501–11. 10.1111/exd.13531 29518287

[107] Poumay Y, Coquette A. Modelling the human epidermis in vitro: tools for basic and applied research. Arch Dermatol Res. 2007;298:361–9. 10.1007/s00403-006-0709-6 PMC1705521 17072628

[108] Prunieras M, Regnier M, Woodley D. Methods for cultivation of keratinocytes with an air-liquid interface. J Invest Dermatol. 1983;81:S28–33. 10.1111/1523-1747.ep12540324 6190962

[109] Handler JS, Green N, Steele RE. Cultures as epithelial models: Porous-bottom culture dishes for studying transport and differentiation. Methods Enzymol. 1989;171:736–44. 10.1016/s0076-6879(89)71040-5 2593858

[110] Voisin C, Aerts C, Jakubczak E, Houdret JL, Tonnel TB. Effects of nitrogen dioxide on alveolar macrophages surviving in the gas phase. A new experimental model for the study of in vitro cytotoxicity of toxic Gases. Bull Eur Physiopathol Respir. 1977;13:137–44. 843644

[111] Lotte C, Patouillet C, Zanini M, Messager A, Roguet R. Permeation and skin absorption: Reproducibility of various industrial reconstructed human skin models. Skin Pharmacol Appl Skin Physiol. 2002;15:18–30. 10.1159/000066679 12476006

[112] Ponec M, Weerheim A, Lankhorst P, Wertz P. New acylceramide in native and reconstructed epidermis. J Invest Dermatol. 2003;120:581–8. 10.1046/j.1523-1747.2003.12103.x 12648220

[113] Thakoersing VS, Gooris GS, Mulder A, Rietveld M, El Ghalbzouri A, Bouwstra JA. Unraveling barrier properties of three different in-house human skin equivalents. Tissue Eng Part C Methods. 2012;18:1–11. 10.1089/ten.TEC.2011.0175 21902617

[114] Thakoersing VS, Van Smeden J, Mulder AA, Vreeken RJ, El Ghalbzouri A, Bouwstra JA. Increased presence of monounsaturated fatty acids in the stratum corneum of human skin equivalents. J Invest Dermatol. 2013;133:59–67. 10.1038/jid.2012.262 22895362

[115] Van Smeden J, Boiten WA, Hankemeier T, Rissmann R, Bouwstra JA, Vreeken RJ. Combined LC/MS-platform for analysis of all major stratum corneum lipids, and the profiling of skin substitutes. Biochim Biophys Acta - Mol Cell Biol Lipids. 2014;1841:70–9. 10.1016/j.bbalip.2013.10.002 24120918

[116] Grice EA, Segre JA. The skin microbiome. Nat Rev Microbiol. 2011;9:244–53. 10.1038/nrmicro2537 PMC3535073 21407241

[117] Byrd AL, Belkaid Y, Segre JA. The human skin microbiome. Nat Rev Microbiol. 2018;16:143–55. 10.1038/nrmicro.2017.157 29332945

[118] Lehtimäki J, Karkman A, Laatikainen T, Paalanen L, Von Hertzen L, Haahtela T, et al. Patterns in the skin microbiota differ in children and teenagers between rural and urban environments. Sci Rep. 2017;7:1–11. 10.1038/srep45651 PMC5374497 28361981

[119] Kim M, Son D, Shin S, Park D, Byun S, Jung E. Protective effects of *Camellia japonica* flower extract against urban air pollutants. BMC Complement Altern Med. 2019;19:30. 10.1186/s12906-018-2405-4 PMC6350298 30691451

[120] Pan TL, Wang PW, Aljuffali IA, Huang CT, Lee CW, Fang JY. The impact of urban particulate pollution on skin barrier function and the subsequent drug absorption. J Dermatol Sci. 2015;78:51–60. 10.1016/j.jdermsci.2015.01.011 25680853

[121] Jin S-P, Li Z, Choi EK, Lee S, Kim YK, Seo EY, et al. Urban particulate matter in air pollution penetrates into the barrier-disrupted skin and produces ROS-dependent cutaneous inflammatory response in vivo. J Dermatol Sci. 2018;91:175–83. 10.1016/j.jdermsci.2018.04.015 29731195

[122] Lee CW, Lin ZC, Hu SCS, Chiang YC, Hsu LF, Lin YC, et al. Urban particulate matter down-regulates filaggrin via COX2 expression/PGE2 production leading to skin barrier dysfunction. Sci Rep. 2016;6:29775. 10.1038/srep27995 PMC4911555 27313009

[123] Piao MJ, Ahn MJ, Kang KA, Ryu YS, Hyun YJ, Shilnikova K, et al. Particulate matter 2.5 damages skin cells by inducing oxidative stress, subcellular organelle dysfunction, and apoptosis. Arch Toxicol. 2018;92:2077–91. 10.1007/s00204-018-2197-9 PMC6002468 29582092

[124] Ryu YS, Kang KA, Piao MJ, Ahn MJ, Yi JM, Bossis G, et al. Particulate matter-induced senescence of skin keratinocytes involves oxidative stress-dependent epigenetic modifications. Exp Mol Med. 2019;51:108. 10.1038/s12276-019-0305-4 PMC6802667 31551408

[125] Nguyen LTH, Nguyen UT, Kim YH, Shin HM, Yang IJ. Astragali Radix and its compound formononetin ameliorate diesel particulate matter-induced skin barrier disruption by regulation of keratinocyte proliferation and apoptosis. J Ethnopharmacol. 2019;228:132–41. 10.1016/j.jep.2018.09.025 30243826

[126] Ha JW, Song H, Hong SS, Boo YC. Marine alga ecklonia cava extract and dieckol attenuate prostaglandin E2 production in HaCaT keratinocytes exposed to airborne particulate matter. Antioxidants. 2019;8:190. 10.3390/antiox8060190 PMC6617419 31234405

[127] Rajagopalan P, Jain AP, Nanjappa V, Patel K, Mangalaparthi KK, Babu N, et al. Proteome-wide changes in primary skin keratinocytes exposed to diesel particulate extract—A role for antioxidants in skin health. J Dermatol Sci. 2018;91:239–49. 10.1016/j.jdermsci.2018.05.003 29857962

[128] Magnani ND, Muresan XM, Belmonte G, Cervellati F, Sticozzi C, Pecorelli A, et al. Skin damage mechanisms related to airborne particulate matter exposure. Toxicol Sci. 2016;149:227–36. 10.1093/toxsci/kfv230 26507108

[129] Iwanaga T, Nioh A, Nicholas R, Kiyokawa H, Akatsuka H. A novel water-in-oil emulsion with a lecithin-modified bentonite prevents skin damage from urban dust and cedar pollen. Int J Cosmet Sci. 2020;42:229–36. 10.1111/ics.12605 PMC7318621 31995229

[130] Kwon K, Park S-H, Han B, Oh S, Lee S, Yoo J, et al. Negative cellular effects of urban particulate matter on human keratinocytes are mediated by P38 MAPK and NF-κB-dependent expression of TRPV 1. Int J Mol Sci. 2018;19:2660. 10.3390/ijms19092660 PMC6163502 30205521

[131] Hieda DS, Anastacio da Costa Carvalho L, Vaz de Mello B, Aparecida de Oliveira E, Romano de Assis S, Wu J, et al. Air particulate matter induces skin barrier dysfunction and water transport alteration on a reconstructed human epidermis model. J Invest Dermatol. 2020;20:31403–2. 10.1016/j.jid.2020.03.971 32339540

[132] Kim H-J, Bae I-H, Son ED, Park J, Cha N, Na H-W, et al. Transcriptome analysis of airborne PM 2.5 -induced detrimental effects on human keratinocytes. Toxicol Lett. 2017;273:26–35. 10.1016/j.toxlet.2017.03.010 28341207

[133] Verdin A, Cazier F, Fitoussi R, Blanchet N, Vié K, Courcot D, et al. An in vitro model to evaluate the impact of environmental fine particles (PM0.3–2.5) on skin damage. Toxicol Lett. 2019;305:94–102. 10.1016/j.toxlet.2019.01.016 30716388

[134] Liao Z, Nie J, Sun P. The impact of particulate matter (PM2.5) on skin barrier revealed by transcriptome analysis: Focusing on cholesterol metabolism. Toxicol Reports. 2020;7:1–9. 10.1016/j.toxrep.2019.11.014 PMC6906712 31867221

[135] Park S-Y, Byun E, Lee J, Kim S, Kim H. Air pollution, autophagy, and skin aging: Impact of particulate matter (PM10) on human dermal fibroblasts. Int J Mol Sci. 2018;19:2727. 10.3390/ijms19092727 PMC6163910 30213068

[136] Wang L, Lee W, Cui YR, Ahn G, Jeon Y-J. Protective effect of green tea catechin against urban fine dust particle-induced skin aging by regulation of NF-κB, AP-1, and MAPKs signaling pathways. Environ Pollut. 2019;252:1318–24. 10.1016/j.envpol.2019.06.029 31252129

[137] Fernando IPS, Jayawardena TU, Kim HS, Vaas APJP, De Silva HIC, Nanayakkara CM, et al. A keratinocyte and integrated fibroblast culture model for studying particulate matter-induced skin lesions and therapeutic intervention of fucosterol. Life Sci. 2019;233:116714. 10.1016/j.lfs.2019.116714 31376370

[138] Fiorito S, Mastrofrancesco A, Cardinali G, Rosato E, Salsano F, Su DS, et al. Effects of carbonaceous nanoparticles from low-emission and older diesel engines on human Skin cells. Carbon N Y. 2011;49:5038–48.

[139] Choi H, Shin DW, Kim W, Doh S-J, Lee SH, Noh M. Asian dust storm particles induce a broad toxicological transcriptional program in human epidermal keratinocytes. Toxicol Lett. 2011;200:92–9. 10.1016/j.toxlet.2010.10.019 21056094

[140] Bae J-E, Choi H, Shin DW, Na H-W, Park NY, Kim JB, et al. Fine particulate matter (PM2.5) inhibits ciliogenesis by increasing SPRR3 expression via c-Jun activation in RPE cells and skin keratinocytes. Sci Rep. 2019;9:399. 10.1038/s41598-019-40670-y PMC6408442 30850686

[141] Kim JH, Son JW, Kim J, Kim MG, Jeong SH, Park TJ, et al. Particulate matter (PM) 2.5 affects keratinocytes via endoplasmic reticulum (ER) stress-mediated suppression of apoptosis. Mol Cell Toxicol. 2020;92:2077–91.

[142] Huang PH, Hu SCS, Yen FL, Tseng CH. Improvement of skin penetration, antipollutant activity and skin hydration of 7,3′,4′-trihydroxyisoflavone cyclodextrin inclusion complex. Pharmaceutics. 2019;11:399. 10.3390/pharmaceutics11080399 PMC6723501 31398912

[143] Lee CW, Chi MC, Peng KT, Chiang YC, Hsu LF, Yan YL, et al. Water-soluble fullerenol C60(OH)36 toward effective anti-air pollution induced by urban particulate matter in HaCaT cell. Int J Mol Sci. 2019;20:4259. 10.3390/ijms20174259 PMC6747515 31480310

[144] Li Q, Kang Z, Jiang S, Zhao J, Yan S, Xu F, et al. Effects of ambient fine particles PM2.5 on human HaCaT cells. Int J Environ Res Public Health. 2017;14:72. 10.3390/ijerph14010072 PMC5295323 28085100

[145] Romani A, Cervellati C, Muresan XM, Belmonte G, Pecorelli A, Cervellati F, et al. Keratinocytes oxidative damage mechanisms related to airbone particle matter exposure. Mech Ageing Dev. 2018;172:86–95. 10.1016/j.mad.2017.11.007 29103985

[146] Scheuplein RJ, Blank IH. Permeability of the skin. Physiol Rev. 1971;51:702–47. 10.1152/physrev.1971.51.4.702 4940637

[147] Madison KC. Barrier function of the skin: “La raison d’être” of the epidermis. J Invest Dermatol. 2003;121:231–41. 10.1046/j.1523-1747.2003.12359.x 12880413

[148] Steven AC, Bisher ME, Roop DR, Steinert PM. Biosynthetic pathways of filaggrin and loricrin--two major proteins expressed by terminally differentiated epidermal keratinocytes. J Struct Biol. 1990;104:150–62. 10.1016/1047-8477(90)90071-j 2088443

[149] Ishida-Yamamoto A, Hohl D, Roop DR, Iizuka H, Eady RA. Loricrin immunoreactivity in human skin: Localization to specific granules (L-granules) in acrosyringia. Arch Dermatol Res. 1993;285:491–8. 10.1007/BF00376822 8274037

[150] Lin TK, Crumrine D, Ackerman LD, Santiago JL, Roelandt T, Uchida Y, et al. Cellular changes that accompany shedding of human corneocytes. J Invest Dermatol. 2012;132:2430–9. 10.1038/jid.2012.173 PMC3447115 22739796

[151] Choi H, Shin JH, Kim ES, Park SJ, Bae I-H, Jo YK, et al. Primary cilia negatively regulate melanogenesis in melanocytes and pigmentation in a human skin model. PLoS One. 2016;11:e0168025. 10.1371/journal.pone.0168025 PMC5152889 27941997

[152] Bäsler K, Bergmann S, Heisig M, Naegel A, Zorn-Kruppa M, Brandner JM. The role of tight junctions in skin barrier function and dermal absorption. J Control Release. 2016;242:105–18. 10.1016/j.jconrel.2016.08.007 27521894

[153] Lehmann AD, Blank F, Baum O, Gehr P, Rothen-Rutishauser BM. Diesel exhaust particles modulate the tight junction protein occludin in lung cells in vitro. Part Fibre Toxicol. 2009;6:26. 10.1186/1743-8977-6-26 PMC2770470 19814802

[154] Zhao R, Guo Z, Zhang R, Deng C, Xu J, Dong W, et al. Nasal epithelial barrier disruption by particulate matter ≤2.5 μm via tight junction protein degradation. J Appl Toxicol. 2018;38:678–87. 10.1002/jat.3573 29235125

[155] Wang T, Wang L, Moreno-Vinasco L, Lang GD, Siegler JH, Mathew B, et al. Particulate matter air pollution disrupts endothelial cell barrier via calpain-mediated tight junction protein degradation. Part Fibre Toxicol. 2012;9:35. 10.1186/1743-8977-9-35 PMC3489700 22931549

[156] The Royal Society and Royal Academy of Engineering. Nanoscience and nanotechnologies: Opportunities and uncertainties. 2004.

[157] Smijs TGM, Bouwstra JA. Focus on skin as a possible port of entry for solid nanoparticles and the toxicological impact. J Biomed Nanotechnol. 2010;6:469–84. 10.1166/jbn.2010.1146 21329042

[158] Larese Filon F, Mauro M, Adami G, Bovenzi M, Crosera M. Nanoparticles skin absorption: New aspects for a safety profile evaluation. Regul Toxicol Pharmacol. 2015;72:310–22. 10.1016/j.yrtph.2015.05.005 25979643

[159] Gläser R, Harder J, Lange H, Bartels J, Christophers E, Schröder J-M. Antimicrobial psoriasin (S100A7) protects human skin from *Escherichia coli* infection. Nat Immunol. 2005;6:57–64. 10.1038/ni1142 15568027

[160] Dürr UHN, Sudheendra US, Ramamoorthy A. LL-37, the only human member of the cathelicidin family of antimicrobial peptides. Biochim Biophys Acta - Biomembr. 2006;1758:1408–25. 10.1016/j.bbamem.2006.03.030 16716248

[161] Schröder JM, Harder J. Antimicrobial skin peptides and proteins. Cell Mol Life Sci. 2006;63:469–86. 10.1007/s00018-005-5364-0 PMC11136068 16416029

[162] Sonnenberg GF, Fouser LA, Artis D. Border patrol: regulation of immunity, inflammation and tissue homeostasis at barrier surfaces by IL-22. Nat Immunol. 2011;12:383–90. 10.1038/ni.2025 21502992

[163] Schieber M, Chandel NS. ROS function in redox signaling and oxidative stress. Curr Biol. 2014;24:R453–62. 10.1016/j.cub.2014.03.034 PMC4055301 24845678

[164] Ursini F, Maiorino M, Forman HJ. Redox homeostasis: The Golden Mean of healthy living. Redox Biol. 2016;8:205–15. 10.1016/j.redox.2016.01.010 PMC4732014 26820564

[165] Kohen R, Nyska A. Oxidation of biological systems: Oxidative stress phenomena, antioxidants, redox reactions, and methods for their quantification. Toxicol Pathol. 2002;30:620–50. 10.1080/01926230290166724 12512863

[166] Gallagher JE, Jackson MA, George MH, Lewtas J. Dose-related differences in DNA adduct levels rodent tissues following skin application of complex mixtures from air pollution sources. Carcinogenesis. 1990;11:63–8. 10.1093/carcin/11.1.63 2403860

[167] Siddens LK, Larkin A, Krueger SK, Bradfield CA, Waters KM, Tilton SC, et al. Polycyclic aromatic hydrocarbons as skin carcinogens: Comparison of benzo [a] pyrene, dibenzo [def,p] chrysene and three environmental mixtures in the FVB/N mouse. Toxicol Appl Pharmacol. 2012;264:377–86. 10.1016/j.taap.2012.08.014 PMC3483092 22935520

[168] Zhen AX, Piao MJ, Hyun YJ, Kang KA, Madushan Fernando PDS, Cho SJ, et al. Diphlorethohydroxycarmalol attenuates fine particulate matter-induced subcellular skin dysfunction. Mar Drugs. 2019;17:95. 10.3390/md17020095 PMC6410332 30717280

[169] Ryu YS, Kang KA, Piao MJ, Ahn MJ, Yi JM, Hyun Y-M, et al. Particulate matter induces inflammatory cytokine production via activation of NFκB by TLR5-NOX4-ROS signaling in human skin keratinocyte and mouse skin. Redox Biol. 2019;21:101080. 10.1016/j.redox.2018.101080 PMC6305701 30584981

[170] Muresan XM, Sticozzi C, Belmonte G, Savelli V, Evelson P, Valacchi G. Modulation of cutaneous scavenger receptor B1 levels by exogenous stressors impairs “in vitro“ wound closure. Mech Ageing Dev. 2018;172:78–85. 10.1016/j.mad.2017.11.006 29102450

[171] Yokota M, Yahagi S, Masaki H. Ethyl 2,4-dicarboethoxy pantothenate, a derivative of pantothenic acid, prevents cellular damage initiated by environmental pollutants through Nrf2 activation. J Dermatol Sci. 2018;92:162–71. 10.1016/j.jdermsci.2018.08.012 30219519

[172] Qiao Y, Li Q, Du H-Y, Wang Q-W, Huang Y, Liu W. Airborne polycyclic aromatic hydrocarbons trigger human skin cells aging through aryl hydrocarbon receptor. Biochem Biophys Res Commun. 2017;488:445–52. 10.1016/j.bbrc.2017.04.160 28526404

[173] Seok JK, Lee J, Kim YM, Boo YC. Punicalagin and (−)-Epigallocatechin-3-Gallate rescue cell viability and attenuate inflammatory responses of human epidermal keratinocytes exposed to airborne particulate matter PM10. Skin Pharmacol Physiol. 2018;31:134–43. 10.1159/000487400 29566388

[174] Lohan SB, Ahlberg S, Mensch A, Höppe D, Giulbudagian M, Calderón M, et al. EPR technology as sensitive method for oxidative stress detection in primary and secondary keratinocytes induced by two selected nanoparticles. Cell Biochem Biophys. 2017;75:359–67. 10.1007/s12013-017-0823-4 28849322

[175] Piao MJ, Kang KA, Zhen AX, Fernando PDSM, Ahn MJ, Koh YS, et al. Particulate matter 2.5 mediates cutaneous cellular injury by inducing mitochondria-associated endoplasmic reticulum stress: Protective effects of ginsenoside Rb1. Antioxidants. 2019;8:383. 10.3390/antiox8090383 PMC6769862 31505827

[176] Chowjarean V, Prueksasit T, Joyjamras K, Chanvorachote P. Isovitexin increases stem cell properties and protects against PM2.5 in keratinocytes. In Vivo (Brooklyn). 2019;33:1833–41. 10.21873/invivo.11676 PMC6899091 31662510

[177] Hu R, Xie X-Y, Xu S-K, Wang Y-N, Jiang M, Wen L-R, et al. PM2.5 exposure elicits oxidative stress responses and mitochondrial apoptosis pathway activation in HaCaT keratinocytes. Chin Med J (Engl). 2017;130:2205–14. 10.4103/0366-6999.212942 PMC5598333 28816208

[178] Kim M, Kim JH, Jeong GJ, Park KY, Lee M, Seo SJ. Particulate matter induces pro-inflammatory cytokines via phosphorylation of p38 MAPK possibly leading to dermal inflammaging. Exp Dermatol. 2019;28:809–15. 10.1111/exd.13943 31001893

[179] Lee C-W, Lin Z-C, Hsu L-F, Fang J-Y, Chiang Y-C, Tsai M-H, et al. Eupafolin ameliorates COX-2 expression and PGE2 production in particulate pollutants-exposed human keratinocytes through ROS/MAPKs pathways. J Ethnopharmacol. 2016;189:300–9. 10.1016/j.jep.2016.05.002 27180879

[180] Kim J, Kim M, Kim J, Lee M, Seo S, Park K. Afzelin suppresses proinflammatory responses in particulate matter-exposed human keratinocytes. Int J Mol Med. 2019;43:2516–22. 10.3892/ijmm.2019.4162 31017255

[181] Lin ZC, Lee CW, Tsai MH, Ko HH, Fang JY, Chiang YC, et al. Eupafolin nanoparticles protect HaCaT keratinocytes from particulate matter-induced inflammation and oxidative stress. Int J Nanomedicine. 2016;11:3907–26. 10.2147/IJN.S109062 PMC4986973 27570454

[182] Zhen AX, Hyun YJ, Piao MJ, Sameera Madushan Fernando PD, Kang KA, Ahn MJ, et al. Eckol inhibits particulate matter 2.5-induced skin keratinocyte damage via MAPK signaling pathway. Mar Drugs. 2019;17:444. 10.3390/md17080444 PMC6723658 31357588

[183] Fernando PDSM, Piao MJ, Zhen AX, Ahn MJ, Yi JM, Choi YH, et al. Extract of *Cornus officinalis* protects keratinocytes from particulate matter-induced oxidative stress. Int J Med Sci. 2020;17:63–70. 10.7150/ijms.36476 PMC6945560 31929739

[184] Zhen AX, Piao MJ, Kang KA, Fernando PDSM, Kang HK, Koh YS, et al. Niacinamide protects skin cells from oxidative stress induced by particulate matter. Biomol Ther (Seoul). 2019;27:562–9. 10.4062/biomolther.2019.061 PMC6824628 31272139

[185] Hyun Y, Piao M, Kang K, Zhen A, Madushan Fernando P, Kang H, et al. Effect of fermented fish oil on fine particulate matter-induced skin aging. Mar Drugs. 2019;17:61. 10.3390/md17010061 PMC6356237 30669248

[186] Hyun YJ, Piao MJ, Kang KA, Ryu YS, Zhen AX, Cho SJ, et al. 3,4-dicaffeoylquinic acid protects human keratinocytes against environmental oxidative damage. J Funct Foods. 2019;52:430–41.

[187] Jang H sung, Lee J eun, Myung C hwan, Park J il, Jo C song, Hwang JS. Particulate matter-induced aryl hydrocarbon receptor regulates autophagy in keratinocytes. Biomol Ther (Seoul). 2019;27:570–6. 10.4062/biomolther.2019.025 PMC6824630 30971064

[188] Øvrevik J, Refsnes M, Låg M, Holme JA, Schwarze PE. Activation of proinflammatory responses in cells of the airway mucosa by particulate matter: Oxidant- and non-oxidant-mediated triggering mechanisms. Biomolecules. 2015;5:1399–440. 10.3390/biom5031399 PMC4598757 26147224

[189] Fang T, Lakey PSJ, Weber RJ, Shiraiwa M. Oxidative potential of particulate matter and generation of reactive oxygen species in epithelial lining fluid. Environ Sci Technol. 2019;53:12784–92. 10.1021/acs.est.9b03823 31560535

[190] Pan C-JG, Schmitz DA, Cho AK, Froines J, Fukuto JM. Inherent redox properties of diesel exhaust particles: Catalysis of the generation of reactive oxygen species by biological reductants. Toxicol Sci. 2004;81:225–32. 10.1093/toxsci/kfh199 15201441

[191] Shinyashiki M, Eiguren-Fernandez A, Schmitz DA, Di Stefano E, Li N, Linak WP, et al. Electrophilic and redox properties of diesel exhaust particles. Environ Res. 2009;109:239–44. 10.1016/j.envres.2008.12.008 19200952

[192] Distefano E, Eiguren-Fernandez A, Delfino RJ, Sioutas C, Froines JR, Cho AK. Determination of metal-based hydroxyl radical generating capacity of ambient and diesel exhaust particles. Inhal Toxicol. 2009;21:731–8. 10.1080/08958370802491433 19242849

[193] Cervellati F, Benedusi M, Manarini F, Woodby B, Russo M, Valacchi G, et al. Proinflammatory properties and oxidative effects of atmospheric particle components in human keratinocytes. Chemosphere. 2020;240:124746. 10.1016/j.chemosphere.2019.124746 31568946

[194] Ntziachristos L, Froines JR, Cho AK, Sioutas C. Relationship between redox activity and chemical speciation of size-fractionated particulate matter. Part Fibre Toxicol. 2007;4. 10.1186/1743-8977-4-5 PMC1899517 17555562

[195] Li N, Sioutas C, Cho A, Schmitz D, Misra C, Sempf J, et al. Ultrafine particulate pollutants induce oxidative stress and mitochondrial damage. Environ Health Perspect. 2003;111:455–60. 10.1289/ehp.6000 PMC1241427 12676598

[196] Xu C, Bailly-Maitre B, Reed JC. Endoplasmic reticulum stress: Cell life and death decisions. J Clin Invest. 2005;115:2656–64. 10.1172/JCI26373 PMC1236697 16200199

[197] Wang G, Yang ZQ, Zhang K. Endoplasmic reticulum stress response in cancer: Molecular mechanism and therapeutic potential. Am J Transl Res. 2010;2:65–74. PMC2826823 20182583

[198] Esser C, Bargen I, Weighardt H, Haarmann-Stemmann T, Krutmann J. Functions of the aryl hydrocarbon receptor in the skin. Semin Immunopathol. 2013;35:677–91. 10.1007/s00281-013-0394-4 23949496

[199] Van Den Bogaard EH, Podolsky MA, Smits JP, Cui X, John C, Gowda K, et al. Genetic and pharmacological analysis identifies a physiological role for the AHR in epidermal differentiation. J Invest Dermatol. 2015;135:1320–8. 10.1038/jid.2015.6 PMC4402116 25602157

[200] Van Den Bogaard EH, Bergboer JGM, Vonk-Bergers M, Van Vlijmen-Willems IMJJ, Hato S V., Van Der Valk PGM, et al. Coal tar induces AHR-dependent skin barrier repair in atopic dermatitis. J Clin Invest. 2013;123:917–27. 10.1172/JCI65642 PMC3561798 23348739

[201] Denison MS, Nagy SR. Activation of the aryl hydrocarbon receptor by structurally diverse exogenous and endogenous chemicals. Annu Rev Pharmacol Toxicol. 2003;43:309–34. 10.1146/annurev.pharmtox.43.100901.135828 12540743

[202] Larigot L, Juricek L, Dairou J, Coumoul X. AhR signaling pathways and regulatory functions. Biochim Open. 2018;7:1–9. 10.1016/j.biopen.2018.05.001 PMC6039966 30003042

[203] Furue M, Takahara M, Nakahara T, Uchi H. Role of AhR/ARNT system in skin homeostasis. Arch Dermatol Res. 2014;306:769–79. 10.1007/s00403-014-1481-7 PMC4220966 24966027

[204] Conney AH. Induction of microsomal enzymes by foreign chemicals and carcinogenesis by polycyclic aromatic hydrocarbons: G. H. A. Clowes memorial lecture. Cancer Res. 1982;42:4875–917. 6814745

[205] Ranjit S, Midde NM, Sinha N, Patters BJ, Rahman MA, Cory TJ, et al. Effect of polyaryl hydrocarbons on cytotoxicity in monocytic cells: Potential role of cytochromes P450 and oxidative stress pathways. PLoS One. 2016;11:e0163827. 10.1371/journal.pone.0163827 PMC5042547 27684561

[206] Shimada T, Fujii-Kuriyama Y. Metabolic activation of polycyclic aromatic hydrocarbons to carcinogens by cytochromes P450 1A1 and 1B1. Cancer Sci. 2004;95:1–6. 10.1111/j.1349-7006.2004.tb03162.x PMC11158916 14720319

[207] Henkler F, Stolpmann K, Luch A. Exposure to polycyclic aromatic hydrocarbons: Bulky DNA adducts and cellular responses. Exp Suppl. 2012;101:107–31. 10.1007/978-3-7643-8340-4_5 22945568

[208] Costa C, Catania S, De Pasquale R, Stancanelli R, Scribano GM, Melchini A. Exposure of human skin to benzo [a]pyrene: Role of CYP1A1 and aryl hydrocarbon receptor in oxidative stress generation. Toxicology. 2010;271:83–6. 10.1016/j.tox.2010.02.014 20307623

[209] Lambeth JD. NOX enzymes and the biology of reactive oxygen. Nat Rev Immunol. 2004;4:181–9. 10.1038/nri1312 15039755

[210] Downing DT, Strauss JS, Pochi PE. Variability in the chemical composition of human skin surface lipids. J Invest Dermatol. 1969;53:322–7. 10.1038/jid.1969.157 5347411

[211] Sousa BC, Pitt AR, Spickett CM. Chemistry and analysis of HNE and other prominent carbonyl-containing lipid oxidation compounds. Free Radic Biol Med. 2017;111:294–308. 10.1016/j.freeradbiomed.2017.02.003 28192230

[212] Pecorelli A, Woodby B, Prieux R, Valacchi G. Involvement of 4-hydroxy-2-nonenal in pollution-induced skin damage. BioFactors. 2019;45:536–47. 10.1002/biof.1513 31087730

[213] Csala M, Kardon T, Legeza B, Lizák B, Mandl J, Margittai É, et al. On the role of 4-hydroxynonenal in health and disease. Biochim Biophys Acta - Mol Basis Dis. 2015;1852:826–38. 10.1016/j.bbadis.2015.01.015 25643868

[214] Negre-Salvayre A, Coatrieux C, Ingueneau C, Salvayre R. Advanced lipid peroxidation end products in oxidative damage to proteins. Potential role in diseases and therapeutic prospects for the inhibitors. Br J Pharmacol. 2008;153:6–20. 10.1038/sj.bjp.0707395 PMC2199390 17643134

[215] Rinnerthaler M, Bischof J, Streubel MK, Trost A, Richter K. Oxidative stress in aging human skin. Biomolecules. 2015;5:545–89. 10.3390/biom5020545 PMC4496685 25906193

[216] Schäfer M, Werner S. The cornified envelope: A first line of defense against reactive oxygen species. J Invest Dermatol. 2011;131:1409–11. 10.1038/jid.2011.119 21673710

[217] Vermeij WP, Alia A, Backendorf C. ROS quenching potential of the epidermal cornified cell envelope. J Invest Dermatol. 2011;131:1435–41. 10.1038/jid.2010.433 21248766

[218] Andersson T, Ertürk Bergdahl G, Saleh K, Magnúsdóttir H, Stødkilde K, Andersen CBF, et al. Common skin bacteria protect their host from oxidative stress through secreted antioxidant RoxP. Sci Rep. 2019;9:1–10. 10.1038/s41598-019-40471-3 PMC6401081 30837648

[219] Haarmann-Stemmann T, Abel J, Fritsche E, Krutmann J. The AhR-Nrf2 pathway in keratinocytes: On the road to chemoprevention. J Invest Dermatol. 2012;132:7–9. 10.1038/jid.2011.359 22158605

[220] Karakoçak BB, Patel S, Ravi N, Biswas P. Investigating the effects of stove emissions on ocular and cancer cells. Sci Rep. 2019;9:1–12. 10.1038/s41598-019-38803-4 PMC6372759 30755694

[221] Nishitoh H. CHOP is a multifunctional transcription factor in the ER stress response. J Biochem. 2012;151:217–9. 10.1093/jb/mvr143 22210905

[222] Pfaffenbach KT, Lee AS. The critical role of GRP78 in physiologic and pathologic stress. Curr Opin Cell Biol. 2011;23:150–6. 10.1016/j.ceb.2010.09.007 PMC3043145 20970977

[223] Matsuyama S, Reed JC. Mitochondria-dependent apoptosis and cellular pH regulation. Cell Death Differ. 2000;7:1155–65. 10.1038/sj.cdd.4400779 11175252

[224] Lamb CA, Yoshimori T, Tooze SA. The autophagosome: Origins unknown, biogenesis complex. Nat Rev Mol Cell Biol. 2013;14:759–74. 10.1038/nrm3696 24201109

[225] Dodig S, Čepelak I, Pavić I. Hallmarks of senescence and aging. Biochem Medica. 2019;29:030501. 10.11613/BM.2019.030501 PMC6610675 31379458

[226] Sadakane K, Ichinose T, Takano H, Yanagisawa R, Inoue K, Kawazato H, et al. Organic chemicals in diesel exhaust particles enhance picryl chloride-induced atopic dermatitis in NC/Nga mice. Int Arch Allergy Immunol. 2013;162:7–15. 10.1159/000350765 23817207

[227] Mastrofrancesco A, Alfè M, Rosato E, Gargiulo V, Beatrice C, Di Blasio G, et al. Proinflammatory effects of diesel exhaust nanoparticles on scleroderma skin cells. J Immunol Res. 2014;2014:138751. 10.1155/2014/138751 PMC4058589 24982919

[228] Fernández JR, Webb C, Rouzard K, Voronkov M, Huber KL, Stock JB, et al. SIG-1273 protects skin against urban air pollution and when formulated in AgeIQ™ night cream anti-aging benefits clinically demonstrated. J Cosmet Dermatol. 2018;18:1366–71. 10.1111/jocd.12825 30456862

[229] Ushio H, Nohara K, Fujimaki H. Effect of environmental pollutants on the production of pro-inflammatory cytokines by normal human dermal keratinocytes. Toxicol Lett. 1999;105:17–24. 10.1016/s0378-4274(98)00379-8 10092052

[230] Huang P-H, Tseng C-H, Lin C-Y, Lee C-W, Yen F-L. Preparation, characterizations and anti-pollutant activity of 7,3′,4′-trihydroxyisoflavone nanoparticles in particulate matter-induced HaCaT keratinocytes. Int J Nanomedicine. 2018;13:3279–93. 10.2147/IJN.S153323 PMC5987860 29910615

[231] Fernando IPS, Jayawardena TU, Sanjeewa KKA, Wang L, Jeon Y-J, Lee WW. Anti-inflammatory potential of alginic acid from Sargassum horneri against urban aerosol-induced inflammatory responses in keratinocytes and macrophages. Ecotoxicol Environ Saf. 2018;160:24–31. 10.1016/j.ecoenv.2018.05.024 29783109

[232] Fernando IPS, Kim H-S, Sanjeewa KKA, Oh J-Y, Jeon Y-J, Lee WW, et al. Inhibition of inflammatory responses elicited by urban fine dust particles in keratinocytes and macrophages by diphlorethohydroxycarmalol isolated from a brown alga Ishige okamurae. ALGAE. 2017;32:261–73.

[233] Ma C, Wang J, Luo J. Activation of nuclear factor kappa B by diesel exhaust particles in mouse epidermal cells through phosphatidylinositol 3-kinase/Akt signaling pathway. Biochem Pharmacol. 2004;67:1975–83. 10.1016/j.bcp.2004.01.023 15130773

[234] Janeway CA. Approaching the asymptote? Evolution and revolution in immunology. Cold Spring Harb Symp Quant Biol. 1989;54 Pt 1:1–13. 24141854

[235] Takeuchi O, Akira S. Pattern recognition receptors and inflammation. Cell. 2010;140:805–20. 10.1016/j.cell.2010.01.022 20303872

[236] Kaisho T, Akira S. Toll-like receptor function and signaling. J Allergy Clin Immunol. 2006;117:979–87. 10.1016/j.jaci.2006.02.023 16675322

[237] Lebre MC, Van Der Aar AMG, Van Baarsen L, Van Capel TMM, Schuitemaker JHN, Kapsenberg ML, et al. Human keratinocytes express functional toll-like receptor 3, 4, 5, and 9. J Invest Dermatol. 2007;127:331–41. 10.1038/sj.jid.5700530 17068485

[238] Yao C, Oh JH, Lee DH, Bae JS, Jin CL, Park CH, et al. Toll-like receptor family members in skin fibroblasts are functional and have a higher expression compared to skin keratinocytes. Int J Mol Med. 2015;35:1443–50. 10.3892/ijmm.2015.2146 25812726

[239] Miller LS, Modlin RL. Human keratinocyte Toll-like receptors promote distinct immune responses. J Invest Dermatol. 2007;127:262–3. 10.1038/sj.jid.5700559 17228303

[240] Kawai T, Akira S. TLR signaling. Cell Death Differ. 2006;13:816–25. 10.1038/sj.cdd.4401850 16410796

[241] Liu T, Zhang L, Joo D, Sun SC. NF-κB signaling in inflammation. Signal Transduct Target Ther. 2017;2:17023. 10.1038/sigtrans.2017.23 PMC5661633 29158945

[242] Smith WL, Michael Garavito R, DeWitt DL. Prostaglandin endoperoxide H synthases (cyclooxygenases)-1 and −2. J Biol Chem. 1996;271:33157–60. 10.1074/jbc.271.52.33157 8969167

[243] Jin Y, Zhu M, Guo Y, Foreman D, Feng F, Duan G, et al. Fine particulate matter (PM2.5) enhances FcεRI-mediated signaling and mast cell function. Cell Signal. 2019;57:102–9. 10.1016/j.cellsig.2019.01.010 30707930

[244] Martinon F, Mayor A, Tschopp J. The inflammasomes: Guardians of the body. Annu Rev Immunol. 2009;27:229–65. 10.1146/annurev.immunol.021908.132715 19302040

[245] Feldmeyer L, Werner S, French LE, Beer H-D. Interleukin-1, inflammasomes and the skin. Eur J Cell Biol. 2010;89:638–44. 10.1016/j.ejcb.2010.04.008 20605059

[246] Keller M, Rüegg A, Werner S, Beer H-D. Active caspase-1 is a regulator of unconventional protein secretion. Cell. 2008;132:818–31. 10.1016/j.cell.2007.12.040 18329368

[247] Zheng R, Tao L, Jian H, Chang Y, Cheng Y, Feng Y, et al. NLRP3 inflammasome activation and lung fibrosis caused by airborne fine particulate matter. Ecotoxicol Environ Saf. 2018;163:612–9. 10.1016/j.ecoenv.2018.07.076 30092543

[248] Du X, Jiang S, Zeng X, Zhang J, Pan K, Song L, et al. Fine particulate matter-induced cardiovascular injury is associated with NLRP3 inflammasome activation in Apo E −/− mice. Ecotoxicol Environ Saf. 2019;174:92–9. 10.1016/j.ecoenv.2019.02.064 30822672

[249] Albanesi C, Scarponi C, Giustizieri ML, Girolomoni G. Keratinocytes in inflammatory skin diseases. Curr Drug Targets Inflamm Allergy. 2005;4:329–34. 10.2174/1568010054022033 16101542

[250] Dieu-Nosjean MC, Massacrier C, Homey B, Vanbervliet B, Pin JJ, Vicari A, et al. Macrophage inflammatory protein 3 alpha is expressed at inflamed epithelial surfaces and is the most potent chemokine known in attracting Langerhans cell precursors. J Exp Med. 2000;192:705–18. 10.1084/jem.192.5.705 PMC2193271 10974036

[251] Ho AW, Kupper TS. T cells and the skin: From protective immunity to inflammatory skin disorders. Nat Rev Immunol. 2019;19:490–502. 10.1038/s41577-019-0162-3 30992525

[252] O’Neill LAJ, Bowie AG. The family of five: TIR-domain-containing adaptors in Toll-like receptor signalling. Nat Rev Immunol. 2007;7:353–64. 10.1038/nri2079 17457343

[253] Mehta V, Vezina CM. Potential protective mechanisms of aryl hydrocarbon receptor (AHR) signaling in benign prostatic hyperplasia. Differentiation. 2011;82:211–9. 10.1016/j.diff.2011.05.011 PMC3179819 21684673

[254] Ito A, Sato T, Iga T, Mori Y. Tumor necrosis factor bifunctionally regulates matrix metalloproteinases and tissue inhibitor of metalloproteinases (TIMP) production by human fibroblasts. FEBS Lett. 1990;269:93–5. 10.1016/0014-5793(90)81127-a 2167246

[255] Bonnans C, Chou J, Werb Z. Remodelling the extracellular matrix in development and disease. Nat Rev Mol Cell Biol. 2014;15:786–801. 10.1038/nrm3904 PMC4316204 25415508

[256] Shin JW, Kwon SH, Choi JY, Na JI, Huh CH, Choi HR, et al. Molecular mechanisms of dermal aging and antiaging approaches. Int J Mol Sci. 2019;20:2126. 10.3390/ijms20092126 PMC6540032 31036793

[257] Shin J-W, Lee H-S, Na J-I, Huh C-H, Park K-C, Choi H-R. Resveratrol inhibits particulate matter-induced inflammatory responses in human keratinocytes. Int J Mol Sci. 2020;21:3446. 10.3390/ijms21103446 PMC7279174 32414118

[258] Lee KE, Ryu JJ, Jo YK, Yeo H, Kang S. 2′-Fucosyllactose attenuates particulate matter-induced inflammation via inhibition of hypoxia-inducible factor in keratinocytes. Biol Pharm Bull. 2019;42:1620–7. 10.1248/bpb.b18-00963 31582650

[259] Lee YH, Park SY, Park JE, Jung BO, Park JE, Park JK, et al. Anti-oxidant activity and dust-proof effect of chitosan with different molecular weights. Int J Mol Sci. 2019;20:3085. 10.3390/ijms20123085 PMC6627310 31238572

[260] Wei Z, Liu X, Ooka M, Zhang L, Song MJ, Huang R, et al. Two-dimensional cellular and three-dimensional bio-printed skin models to screen topical-use compounds for irritation potential. Front Bioeng Biotechnol. 2020;8:109. 10.3389/fbioe.2020.00109 PMC7046801 32154236

[261] Mieremet A, Rietveld M, Absalah S, Van Smeden J, Bouwstra JA, El Ghalbzouri A. Improved epidermal barrier formation in human skin models by Chitosan modulated dermal matrices. PLoS One. 2017;12:e0174478. 10.1371/journal.pone.0174478 PMC5363943 28333992

[262] Mieremet A, Vázquez García A, Boiten W, van Dijk R, Gooris G, Bouwstra JA, et al. Human skin equivalents cultured under hypoxia display enhanced epidermal morphogenesis and lipid barrier formation. Sci Rep. 2019;9:7811. 10.1038/s41598-019-44204-4 PMC6534609 31127151

[263] Pupovac A, Senturk B, Griffoni C, Maniura-Weber K, Rottmar M, McArthur SL. Toward immunocompetent 3D skin models. Adv Healthc Mater. 2018;7:e1701405. 10.1002/adhm.201701405 29542274

[264] Miyazaki H, Tsunoi Y, Akagi T, Sato S, Akashi M, Saitoh D. A novel strategy to engineer pre-vascularized 3-dimensional skin substitutes to achieve efficient, functional engraftment. Sci Rep. 2019;9:7797. 10.1038/s41598-019-44113-6 PMC6534548 31127144

[265] Randall MJ, Jüngel A, Rimann M, Wuertz-Kozak K. Advances in the biofabrication of 3D skin in vitro: Healthy and pathological models. Front Bioeng Biotechnol. 2018;6:154. 10.3389/fbioe.2018.00154 PMC6220074 30430109

[266] Stefaniak AB, Harvey CJ. Dissolution of materials in artificial skin surface film liquids. Toxicol Vitr. 2006;20:1265–83. 10.1016/j.tiv.2006.05.011 16860531

[267] Wertz PW. Human synthetic sebum formulation and stability under conditions of use and storage. Int J Cosmet Sci. 2009;31:21–5. 10.1111/j.1468-2494.2008.00468.x 19134124

[268] De Vuyst E, Salmon M, Evrard C, de Rouvroit CL, Poumay Y. Atopic Dermatitis studies through in vitro models. Front Med. 2017;14:119. 10.3389/fmed.2017.00119 PMC5523664 28791291

[269] Löwa A, Jevtić M, Gorreja F, Hedtrich S. Alternatives to animal testing in basic and preclinical research of atopic dermatitis. Exp Dermatol. 2018;27:476–83. 10.1111/exd.13498 29356091

[270] Nakamura M, Haarmann-Stemmann T, Krutmann J, Morita A. Alternative test models for skin ageing research. Exp Dermatol. 2018;27:495–500. 10.1111/exd.13519 29478289

[271] Platt SM, El Haddad I, Pieber SM, Zardini AA, Suarez-Bertoa R, Clairotte M, et al. Gasoline cars produce more carbonaceous particulate matter than modern filter-equipped diesel cars. Sci Rep. 2017;7:4926. 10.1038/s41598-017-03714-9 PMC5509693 28706240

[272] Mannucci PM, Franchini M. Health effects of ambient air pollution in developing countries. Int J Environ Res Public Health. 2017;14:1048. 10.3390/ijerph14091048 PMC5615585 28895888

[273] Soeur J, Belaïdi JP, Chollet C, Denat L, Dimitrov A, Jones C, et al. Photo-pollution stress in skin: Traces of pollutants (PAH and particulate matter) impair redox homeostasis in keratinocytes exposed to UVA1. J Dermatol Sci. 2017;86:162–9. 10.1016/j.jdermsci.2017.01.007 28153538

[274] Burke KE, Wei H. Synergistic damage by UVA radiation and pollutants Carcinogenesis by BaP. Toxicol Ind Health. 2009;25:219–24. 10.1177/0748233709106067 19651790

